# Supplementary material for: Production of MCM-41 Nanoparticles with Control of Particle Size and Structural Properties: Optimizing Operational Conditions during Scale-Up
Source: Int J Mol Sci. 2020 Oct 24;21(21):7899. doi: 10.3390/ijms21217899 (PMC7662541; doi:10.3390/ijms21217899)
Supplement: Supplementary file 1 [file ijms-21-07899-s001.pdf]

Production of MCM-41 nanoparticles with control of particle size and structural properties: optimizing operational conditions during scale-up

Rafael R. Castillo[1,2,3], Lorena de la Torre[4], Félix García-Ochoa[4,a], Miguel Ladero[4,a,*], María Vallet-Regí[1,2,3,a,*]

[1] Department of Chemistry in Pharmaceutical Sciences. Faculty of Pharmacy. Universidad Complutense de Madrid. 28040, Madrid, Spain. rafcas01@ucm.es; vallet@ucm.es

[2] Biomedical Research Centre Network (CIBER). Av. Monforte de Lemos 3-5, 28029, Madrid, Spain.

[3] imas12 Research Institute, Hospital 12 de Octubre, Av. Córdoba s/n, 28041, Madrid, Spain

[4] Department of Chemical and Materials Engineering. Faculty of Chemistry. Universidad Complutense de Madrid. 28040, Madrid, Spain. lorenadelatorresierra@gmail.com; fgochoa@ucm.es; mladerog@ucm.es

[a] These authors share senior authorship

* Correspondence: mladerog@ucm.es; vallet@ucm.es

Supplementary Material.

Summary of synthesis conditions. S2

Dynamic Light Scattering and z-potential measurements for MCM-41 MSNs. S4

Representative TEM images for MCM-41 MSNs. S8

Particle size distribution and Feret diameter obtained from TEM micrographs. S14

Infrared spectra for extracted MCM-41 MSNs. S22

Small Angle X-ray diffraction spectra of extracted MCM-41 MSNs. S24

N_2 adsorption isotherms of extracted MCM-41 MSNs. S26

Pore diameters obtained for extracted MCM-41 MSNs. S28

Calculated surface areas and pore diameters for extracted MCM-41 MSNs. S30

Summary of synthesis conditions.

Sample	Temp. (ºC)	Maturation (min)	Volume (L)	TEOS (mL)	CTAB (g)	Stirring (rpm)	Stirrer	TEOS purity	TEOS (mL/min)	Hydrodynamic Diameter(nm)	Standard deviation
CTAB/TEOS (1L round bottom spherical flask)											
D3	80	60	0.5	5	0.5	800	Magnet	99.99%	0.5	1153.0	94.21
D5	80	60	0.5	10	0.5	800	Magnet	99.99%	0.5	970.5	21.91
D7	80	60	0.5	5	1	800	Magnet	99.99%	0.5	242.2	9.342
Temperature (1L round bottom spherical flask)											
C1	50	60	0.5	5	1	800	Magnet	>98%	0.5	249.6	4.251
C2	60	60	0.5	5	1	800	Magnet	>98%	0.5	248.4	4.137
C3	70	60	0.5	5	1	800	Magnet	>98%	0.5	291.9	12.69
C5	80	60	0.5	5	1	800	Magnet	>98%	0.5	240.2	3.331
C6	90	60	0.5	5	1	800	Magnet	>98%	0.5	741.1	19.14
Stirrer (5 L cylindrical reactor)											
B1	80	60	4	40	8	400	Elephant ear	>98%	4	294.7	17.60
B2	80	60	4	40	8	400	Paddle	>98%	4	191.8	5.492
B3	80	60	4	40	8	400	Paddle	>98%	4	270.1	5.074
B4	80	60	4	40	8	400	Rushton	>98%	4	216.3	7.228
B5	80	60	4	40	8	400	Anchor	>98%	4	180.5	1.906
B6	80	60	4	40	8	400	Anchor	>98%	4	384.5	16.22
Stirring speed (Rushton Turbine, 5L cylindrical reactor)											
345.2	60	60	4	40	8	400	Rushton	>98%	4	230.9	1.82
345.4	60	60	4	40	8	400	Rushton	>98%	4	175.8	4.571
389.1	60	60	4	40	8	650	Rushton	>98%	4	281.6	5.477
349.2	60	60	4	40	8	650	Rushton	>98%	4	272.4	4.563

Sample	Temp. (ºC)	Maturation (min)	Volume (L)	TEOS	CTAB (g)	Stirring (rpm)	Stirrer	TEOS	TEOS (mL/min)	Hydrodynamic Diameter(nm)	Standard deviation
TEOS addition speed (5 L cylindrical reactor)											
345.3	60	60	4	40	8	650	Rushton	>98%	2	183.7	2.360
349.2	60	60	4	40	8	650	Rushton	>98%	4	272.4	4.563
349.1	60	60	4	40	8	650	Rushton	>98%	6	295.6	4.396
345.7	60	60	4	40	8	650	Rushton	>98%	10.7	303.4	2.949
345.6	60	60	4	40	8	650	Rushton	>98%	290	822.8	20.15
Maturation time (5L cylindrical reactor)											
		5								297.4	8.132
359.2	60	30	4	40	8	650	Rushton	>98%	4	256.2	6.690
		60								214.1	3.690

Table S1: Synthesis conditions for MCM-41 like Mesoporous Silica Nanoparticles. All syntheses were accomplished employing a solution of CTAB in water set to a pH ≈ 11.8 with NaOH; this solution was allowed to stabilize for at least 45 minutes prior to the addition of TEOS, which was added dropwise with the aid of either an addition funnel or a syringe pump. In all examples, maturation time started when TEOS addition was completed. When the whole process was completed, the resulting suspensions were collected, cooled in an ice bath for 10 minutes and centrifuged (10000 rpm, 10 minutes), discarding the supernatant. Then, the particles were washed twice with water and twice with EtOH to finally, be dispersed in EtOH and stored refrigerated. Removal of the CTAB template from the mesopores was achieved after two reflux cycles (2h) of acidic exchange extraction with a 10mg/mL solution of NH_4NO_3 in EtOH/H_2O (95:5) followed thorough EtOH washing. When necessary the particles were vacuum dried at 37ºC for 24h. Stirrers employed were either a 40 mm diameter oval magnet or a 60 mm Rushton turbine unless otherwise noted.

Dynamic Light Scattering and z-potential measurements for MCM-41 MSNs.

Hydrodynamic size and z-potential measurements for prepared MCM-41 MSNs. All measurements were recorded in EtOH upon surfactant removal. The represented diameter corresponds to an average of 5 measurements. Both z-potential and hydrodynamic diameter of nanoparticles were measured by means of a Zetasizer Nano ZS (Malvern Instruments) equipped with a 633 nm laser.

Sample — ***Dynamic Light Scattering diameter (nm)*** — ***ζ-potential (mV)***

D3

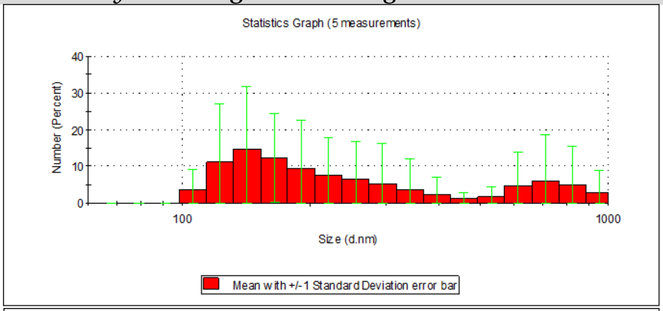

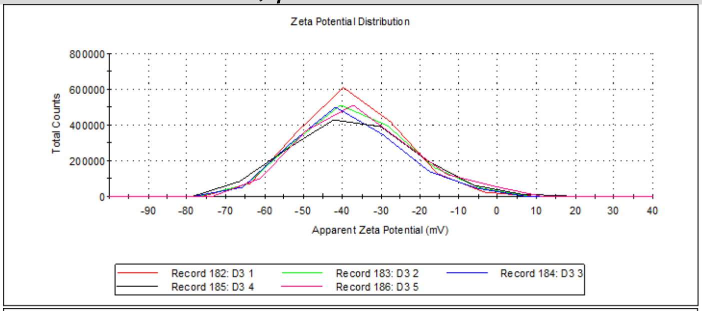

D5

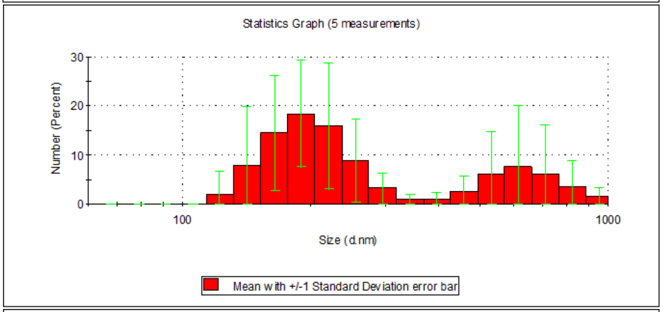

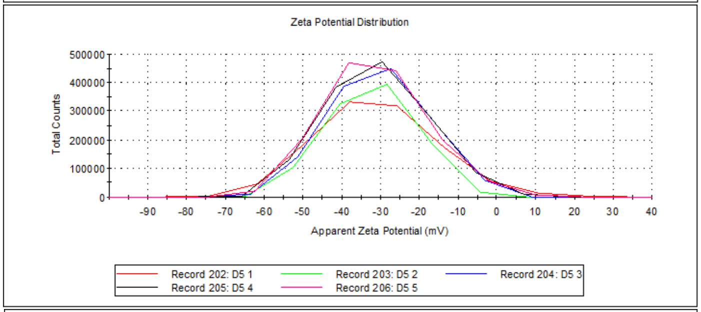

D7

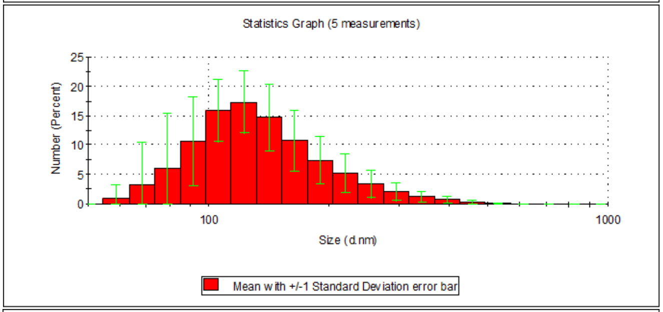

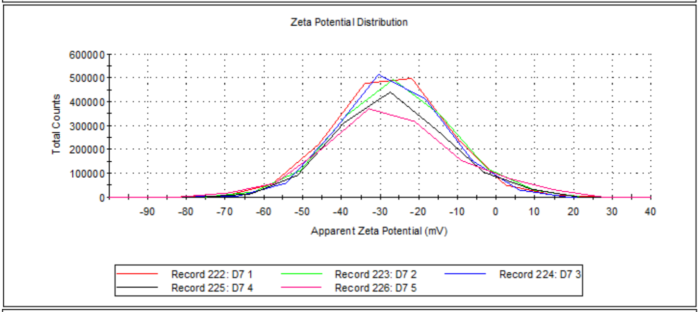

C1

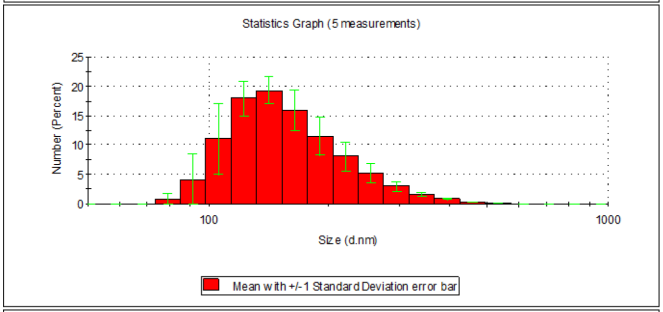

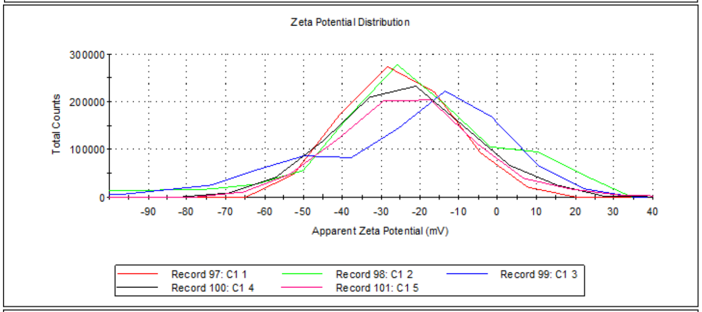

C2

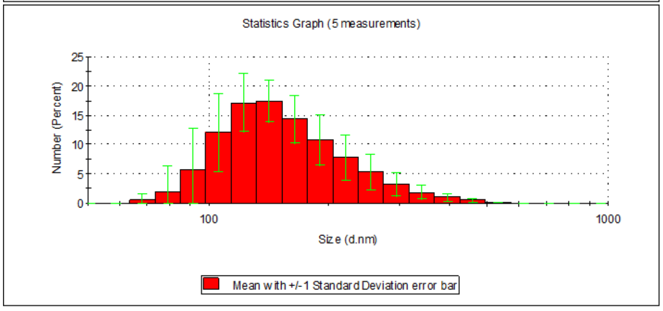

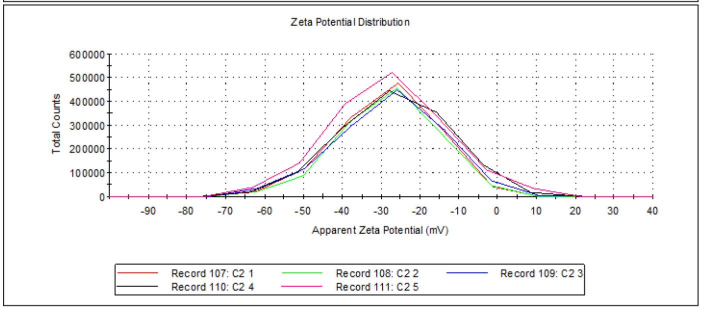

C3

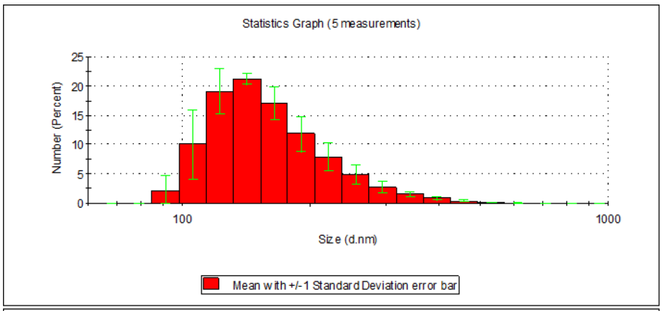

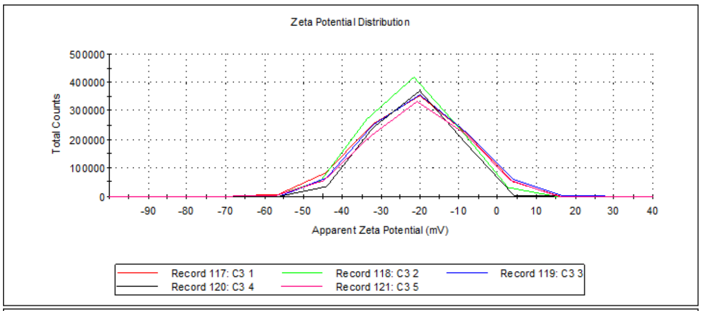

C5

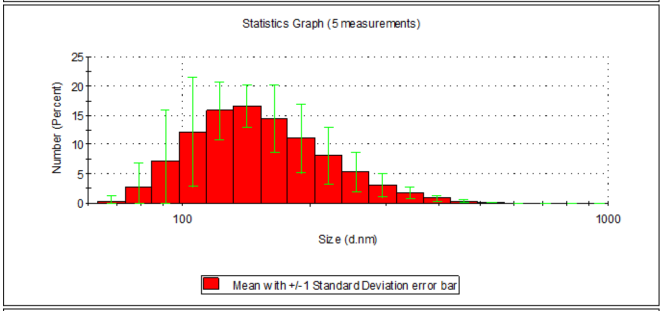

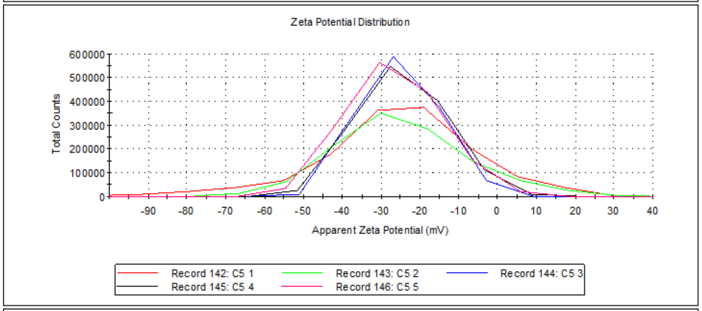

C6

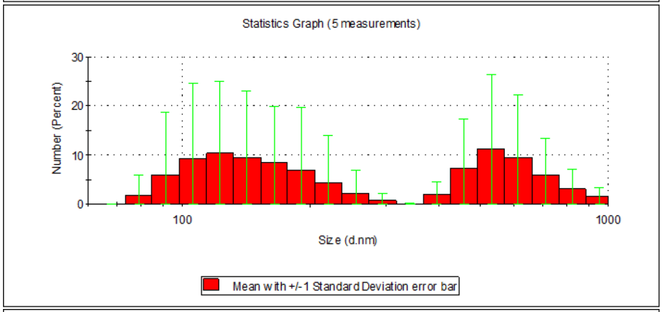

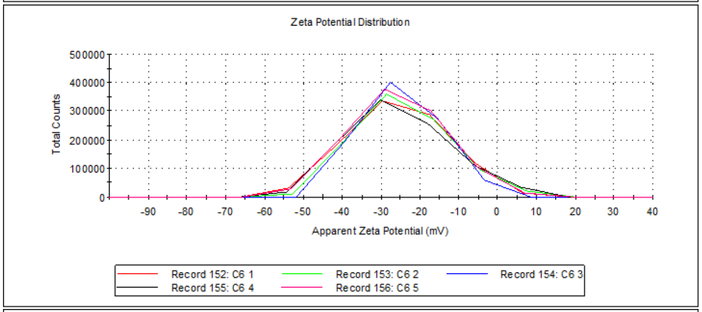

B1

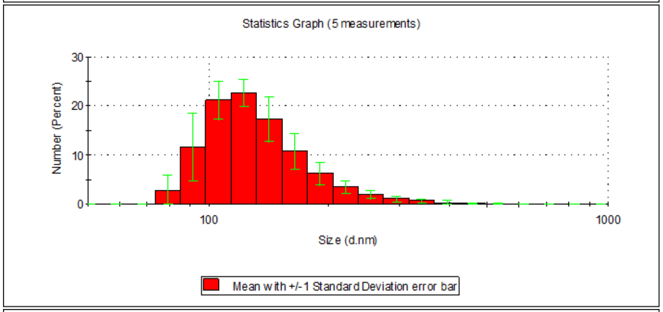

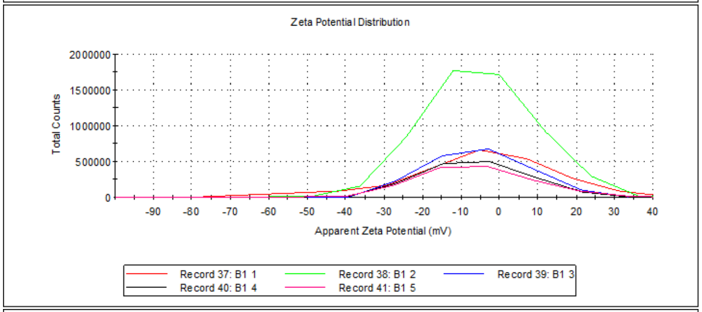

B2

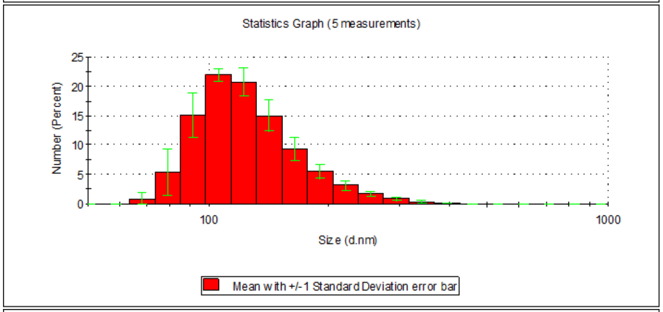

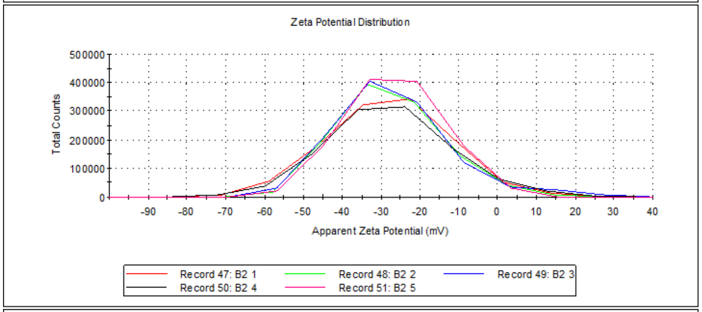

B3

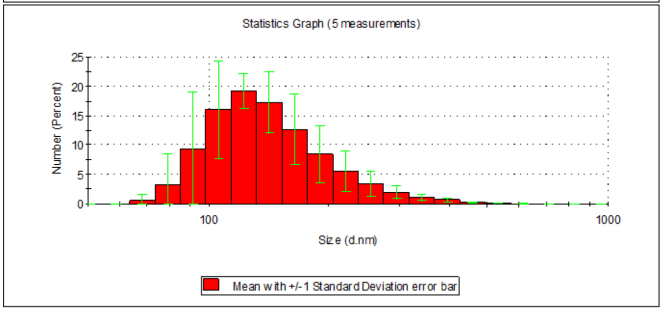

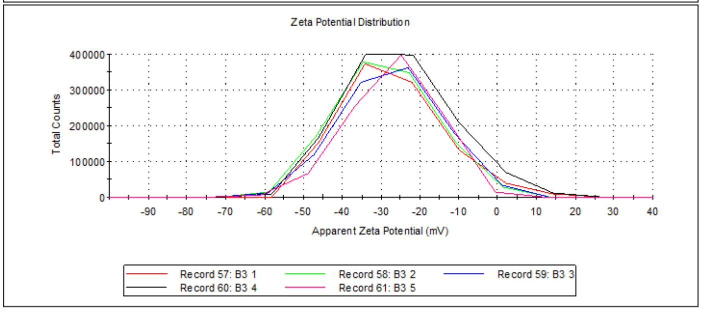

B4

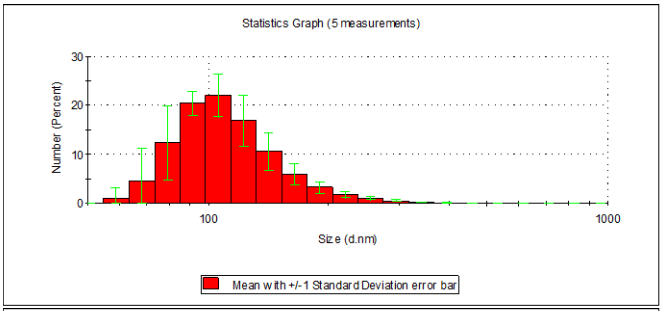

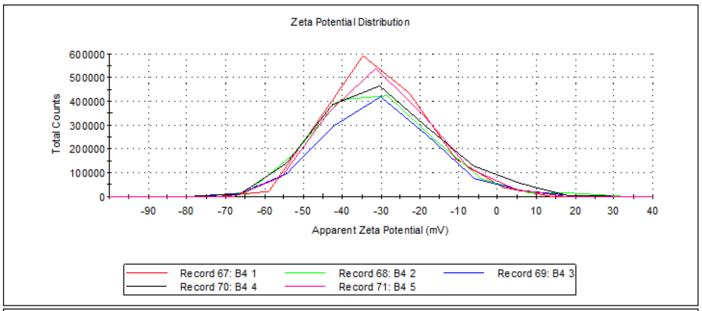

B5

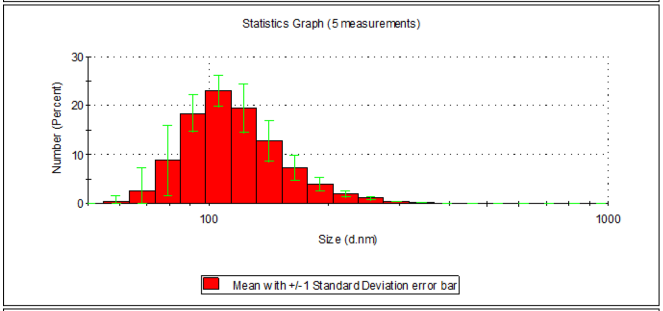

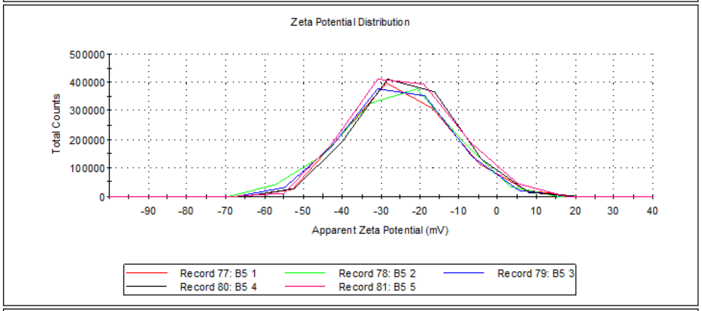

B6

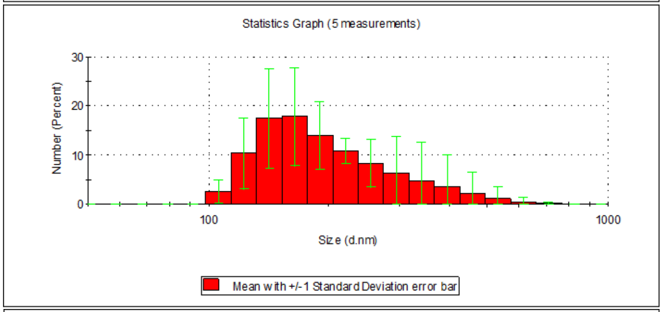

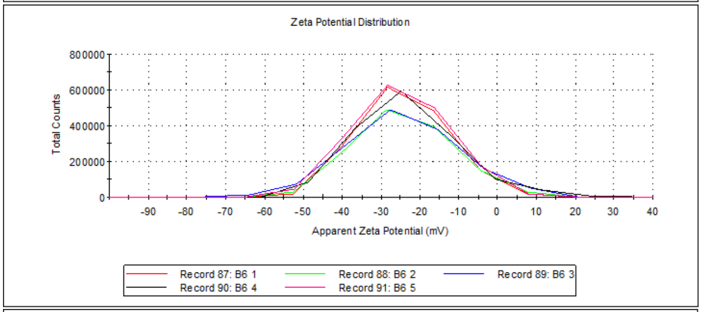

345.2

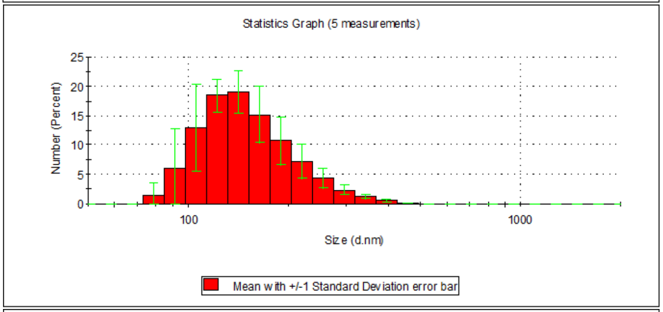

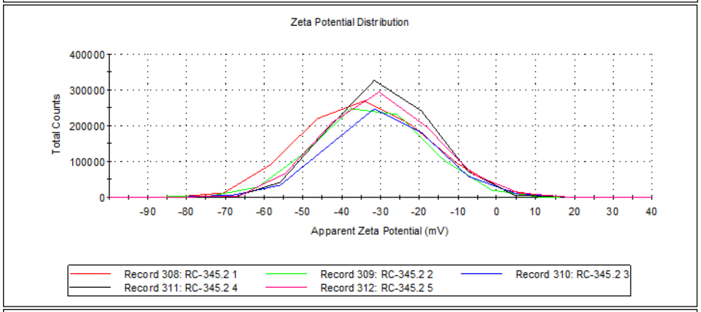

345.4

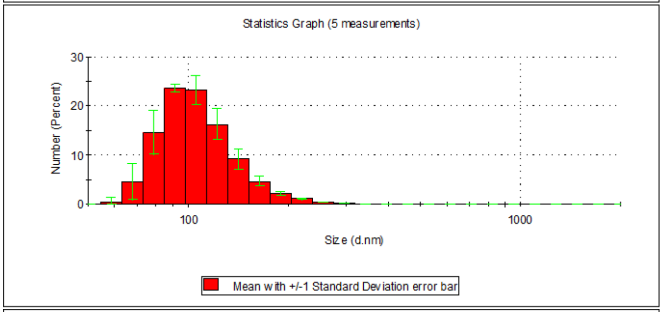

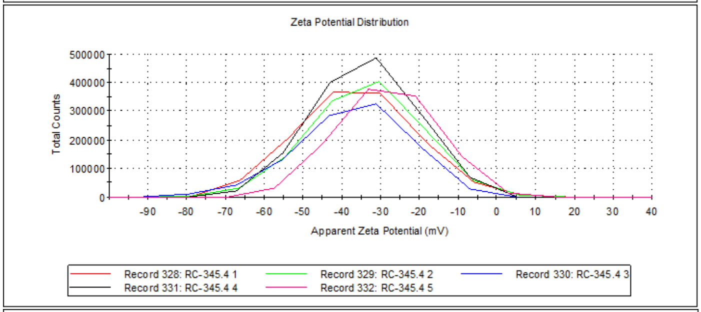

389.1

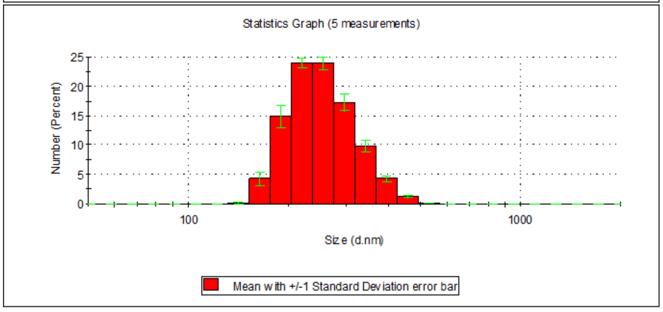

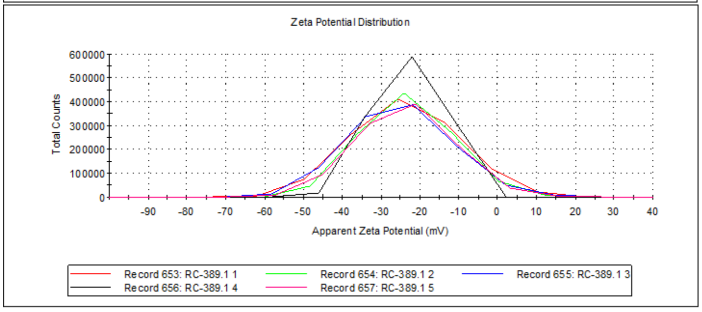

345.3

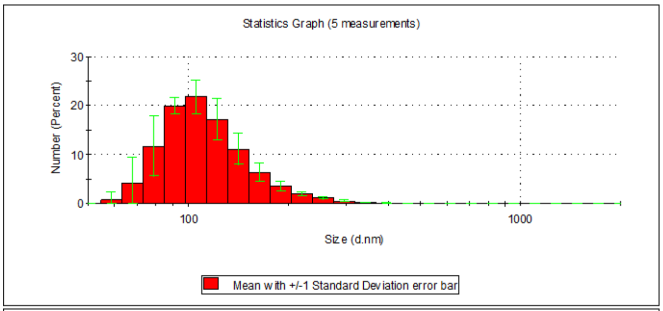

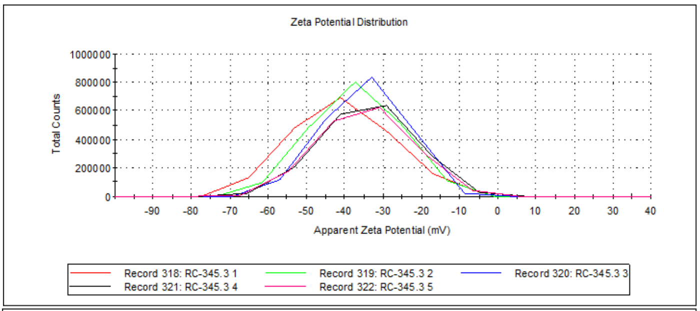

349.2

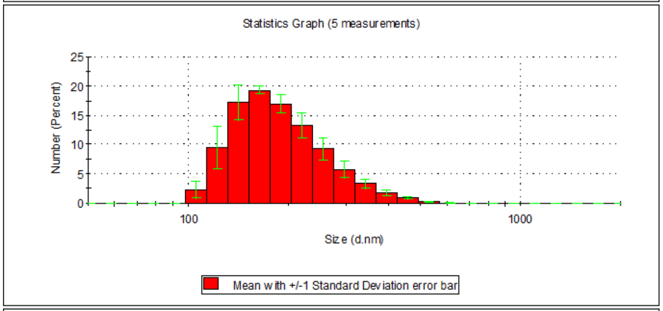

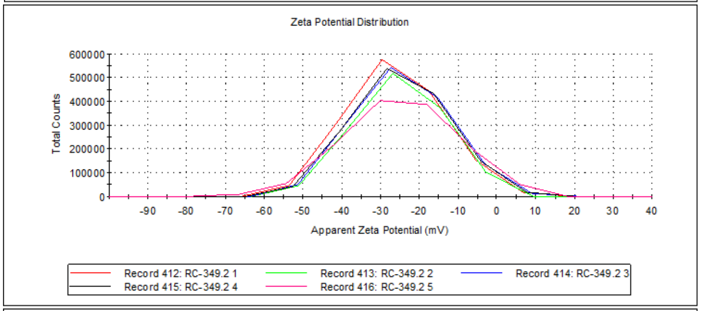

349.1

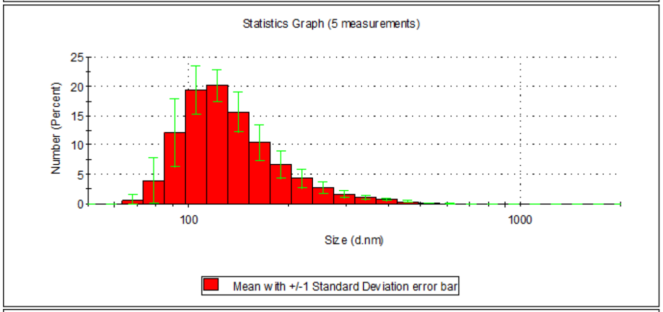

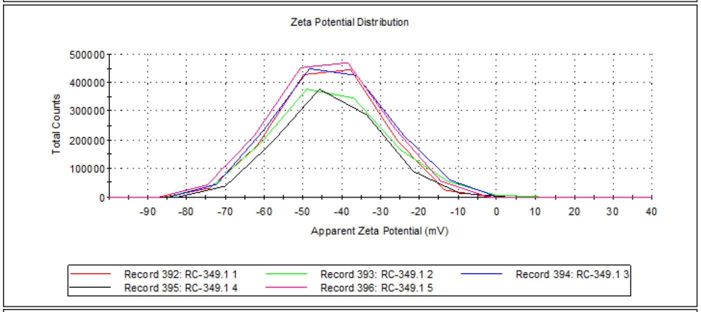

345.7

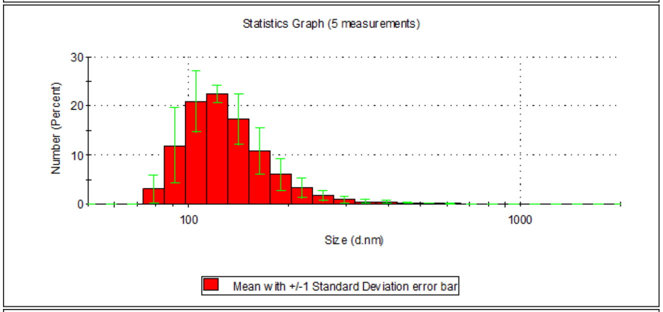

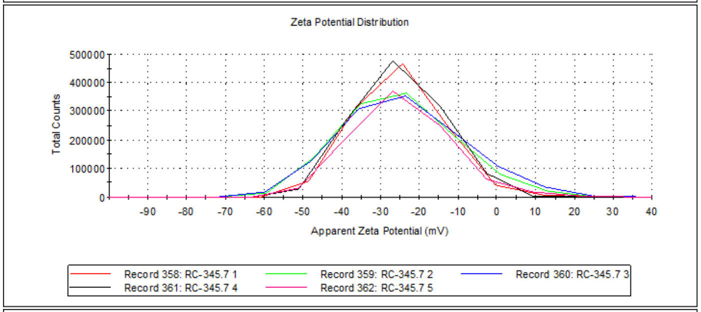

345.6

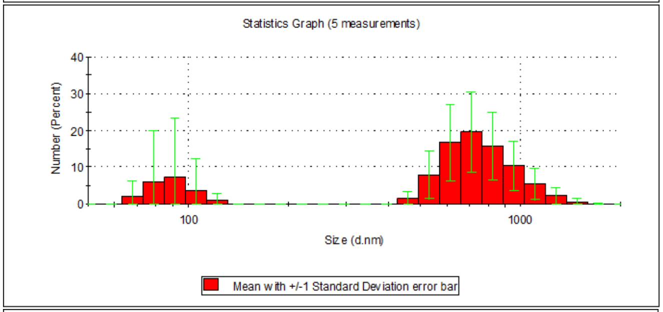

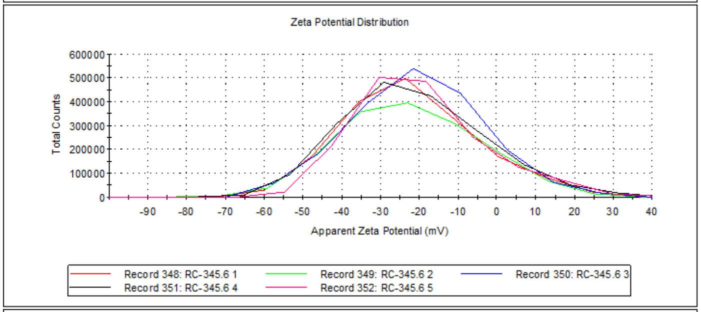

359.2
5min

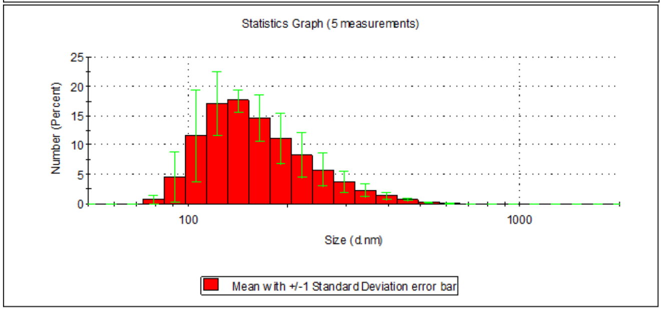

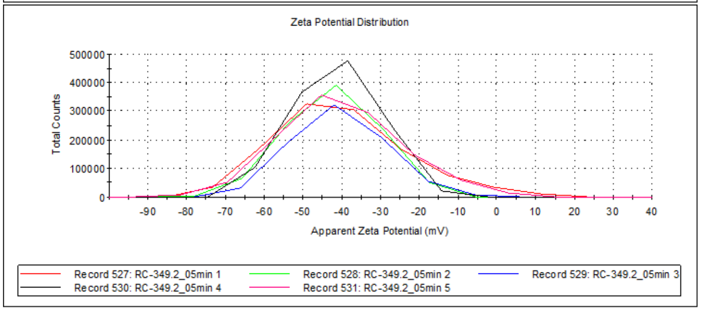

359.2
30min

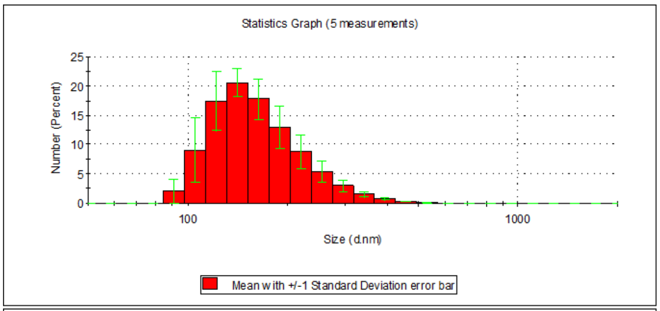

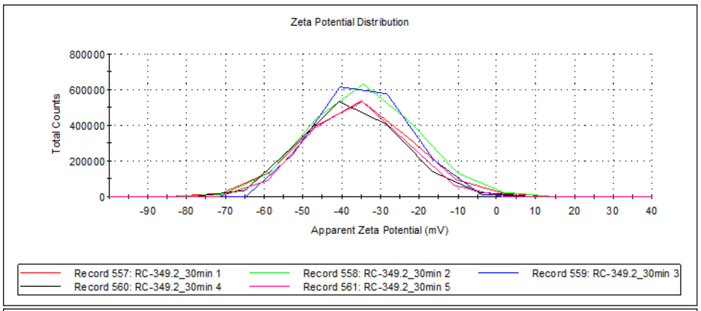

359.2
60min

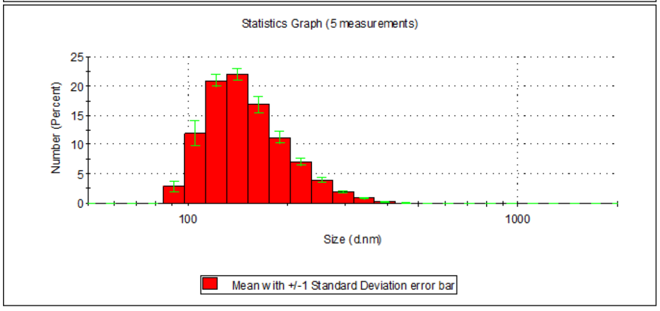

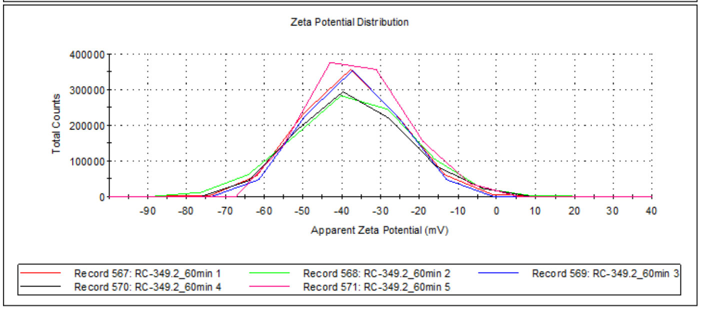

Representative TEM images for MCM-41 MSNs.

The morphology of particles was analyzed by transmission electron microscopy (TEM), which was carried on either a JEOL JEM 2100 or JEM 1400 microscopes equipped with a charge-coupled device (CCD) camera (KeenView Camera). Samples were prepared onto treated Cu grids to which were added a drop of the corresponding suspension of MSNs in EtOH.

Sample | *Representative TEM images*

D3

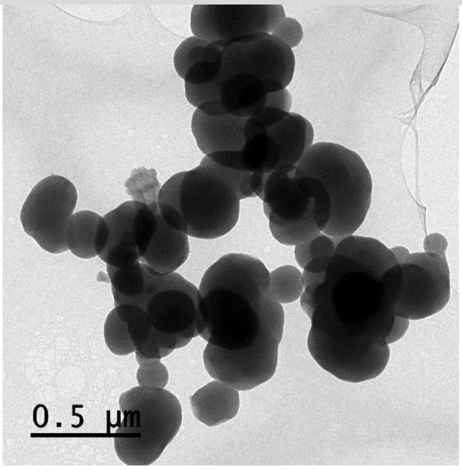

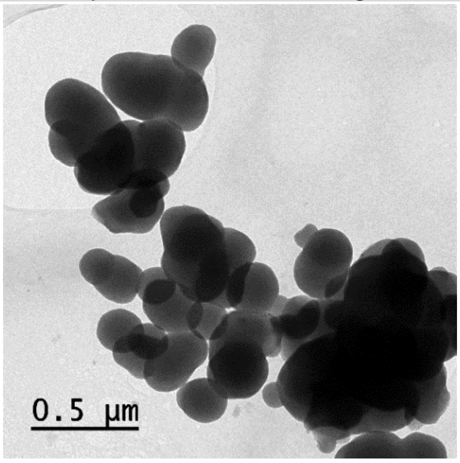

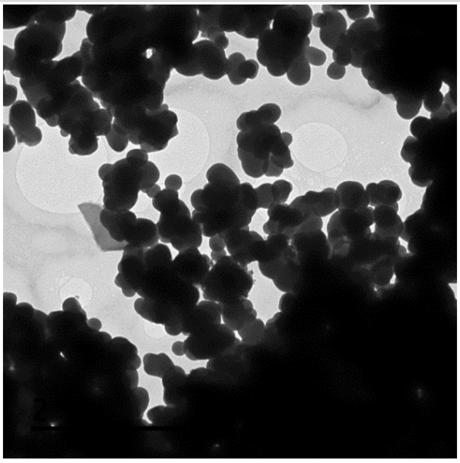

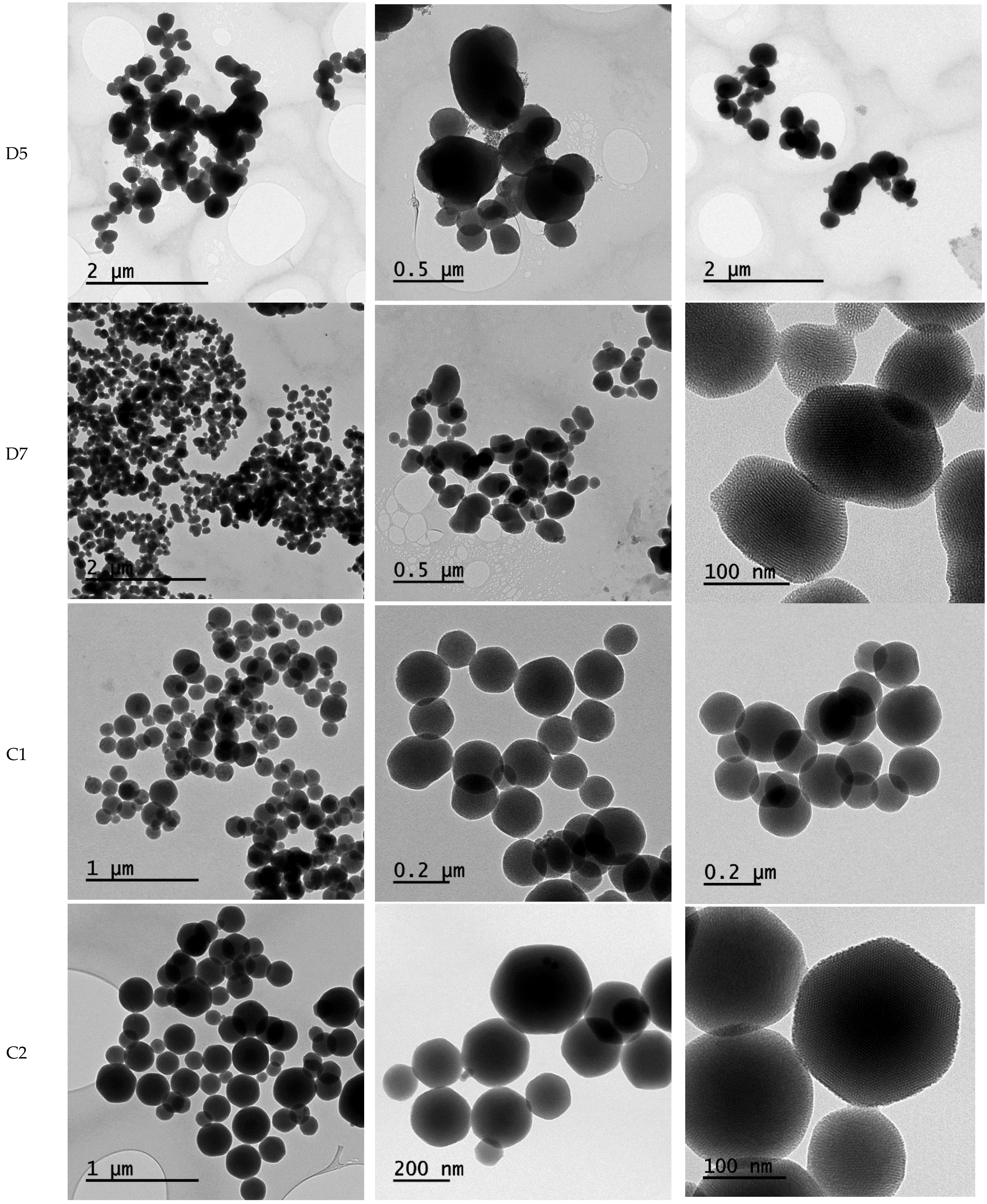
D5
2 µm
0.5 µm
2 µm
D7
2 µm
0.5 µm
100 nm
C1
1 µm
0.2 µm
0.2 µm
C2
1 µm
200 nm
100 nm

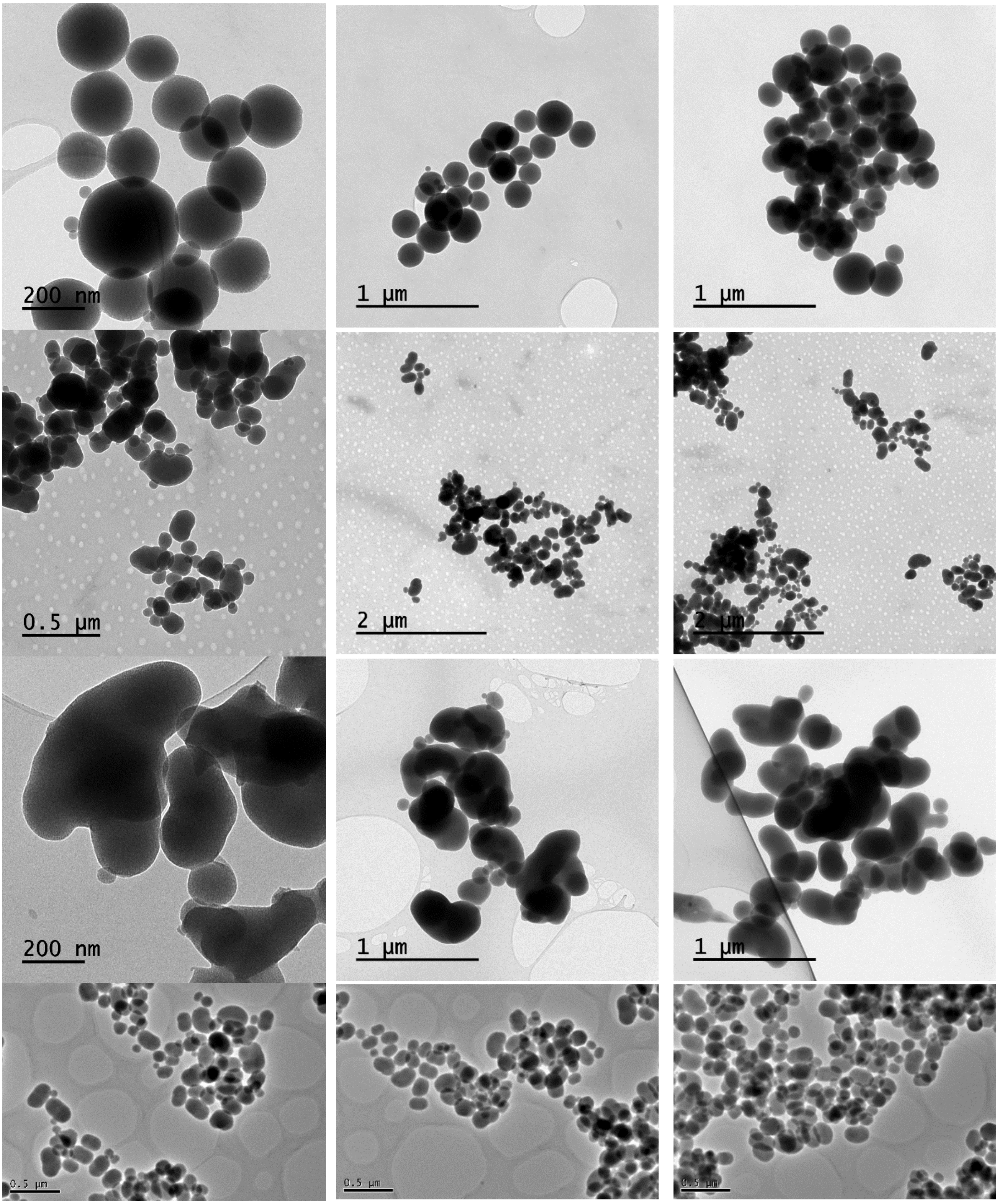

C3

C5

C6

B1

B2

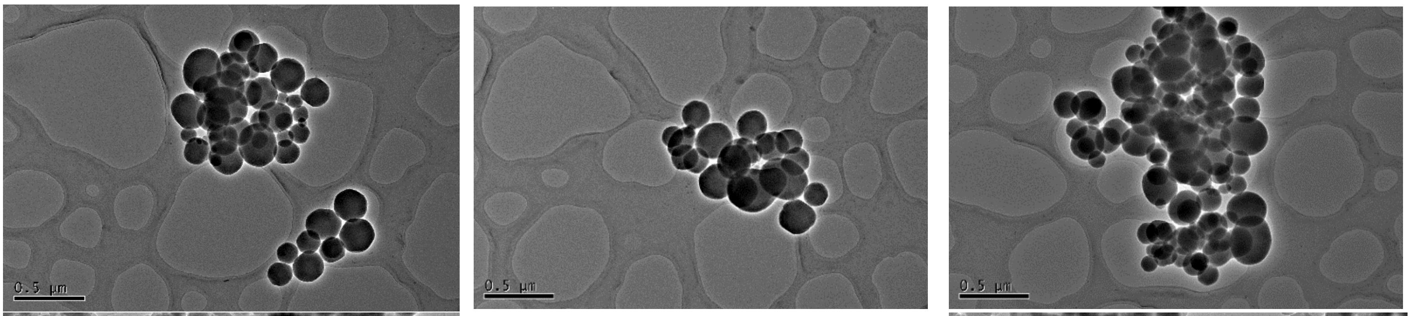
0.5 µm
0.5 µm
0.5 µm

B3

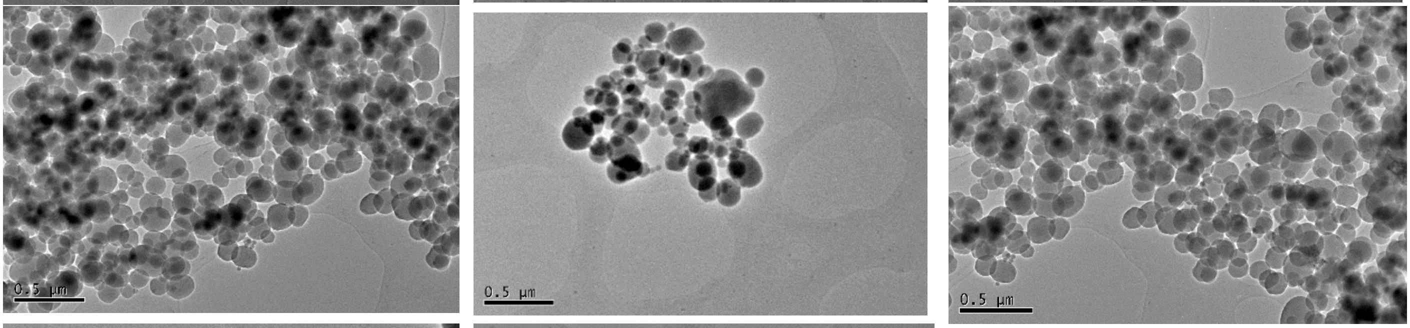
0.5 µm
0.5 µm
0.5 µm

B4

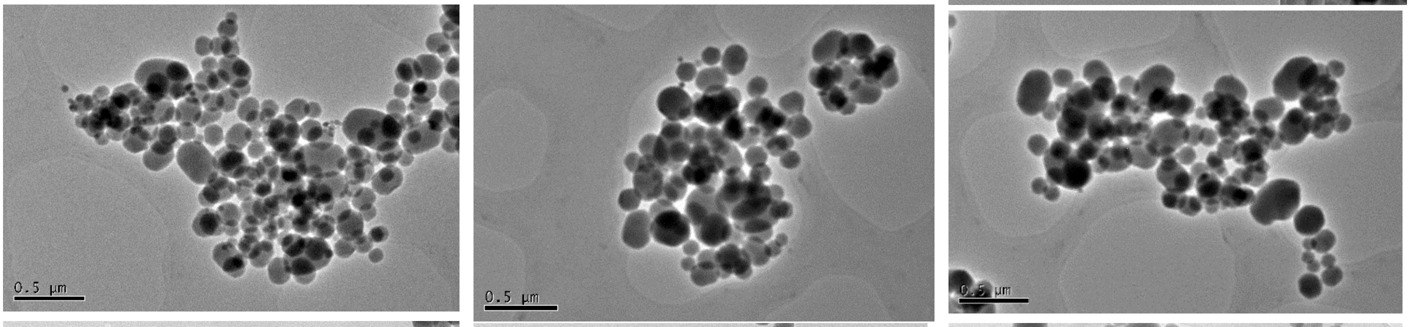
0.5 µm
0.5 µm
0.5 µm

B5

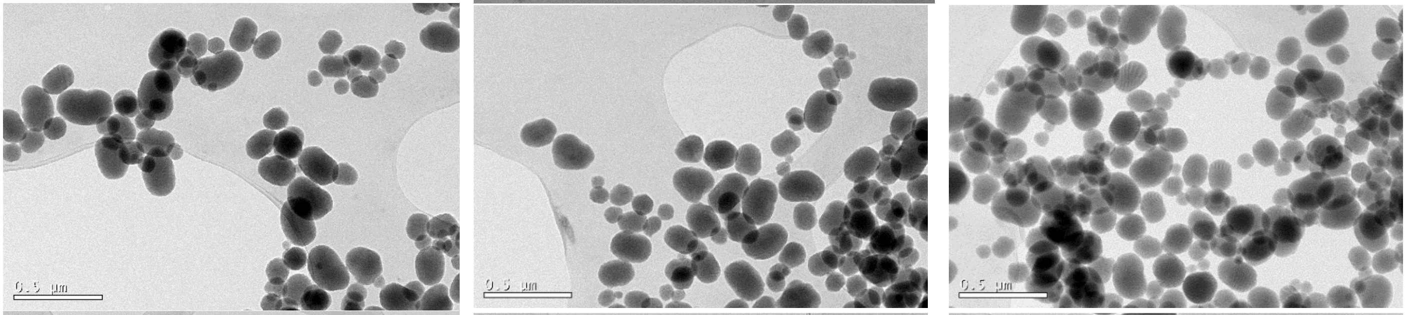
0.5 µm
0.5 µm
0.5 µm

B6

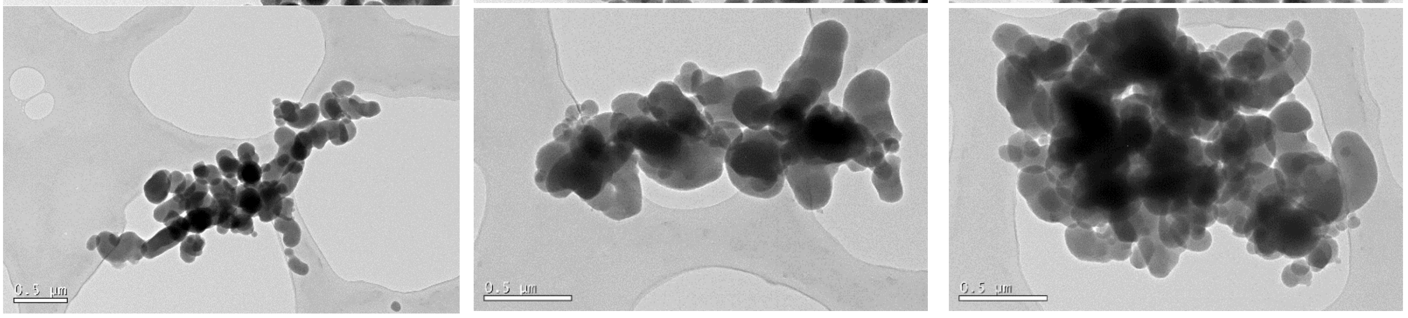
0.5 µm
0.5 µm
0.5 µm

345.2

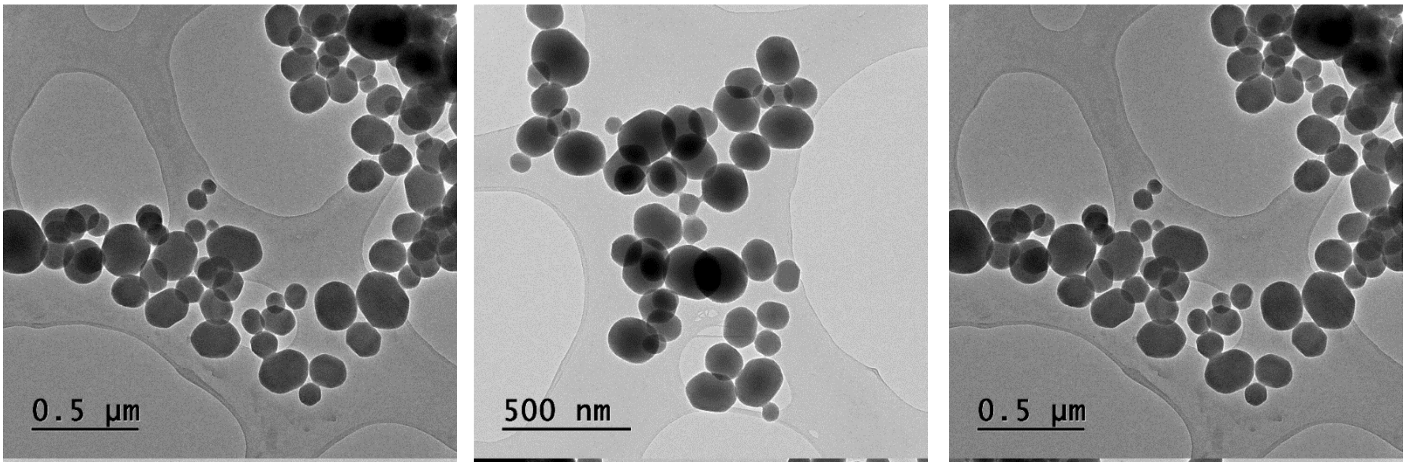

345.4

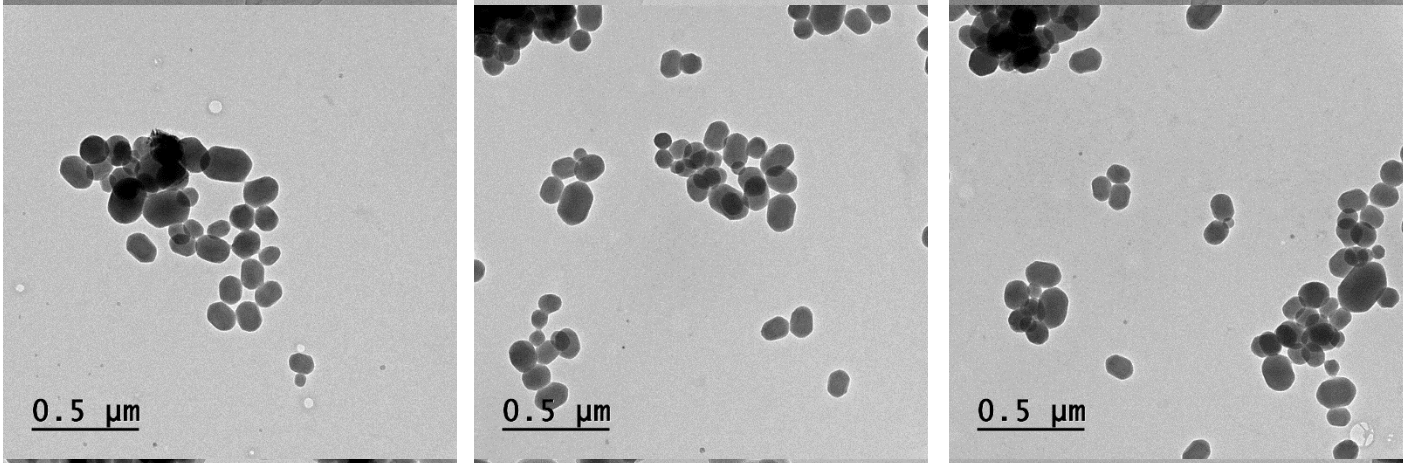

389.1

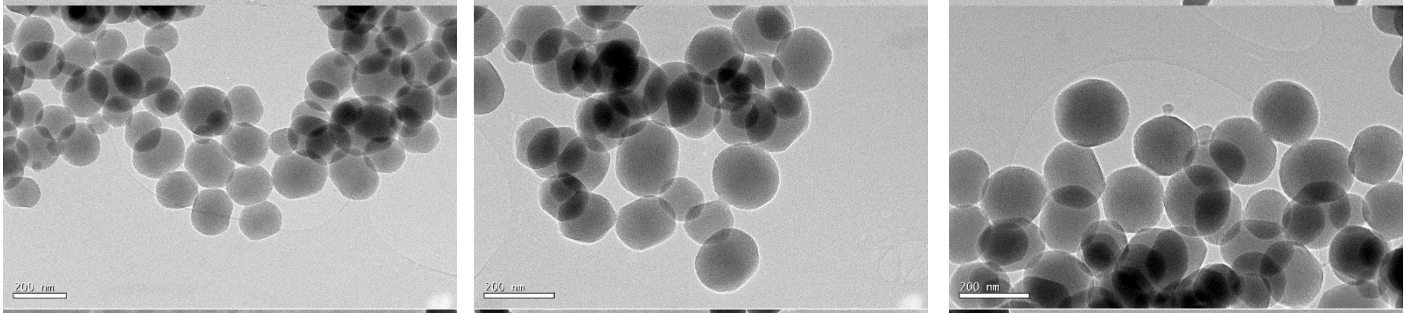

345.3

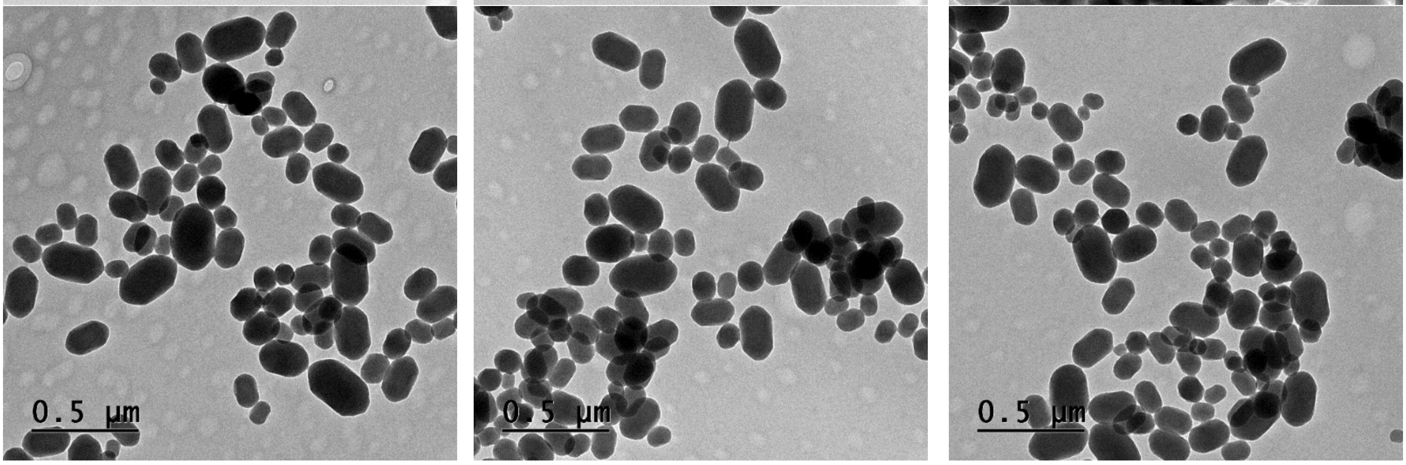

349.2

349.1

345.7

345.6

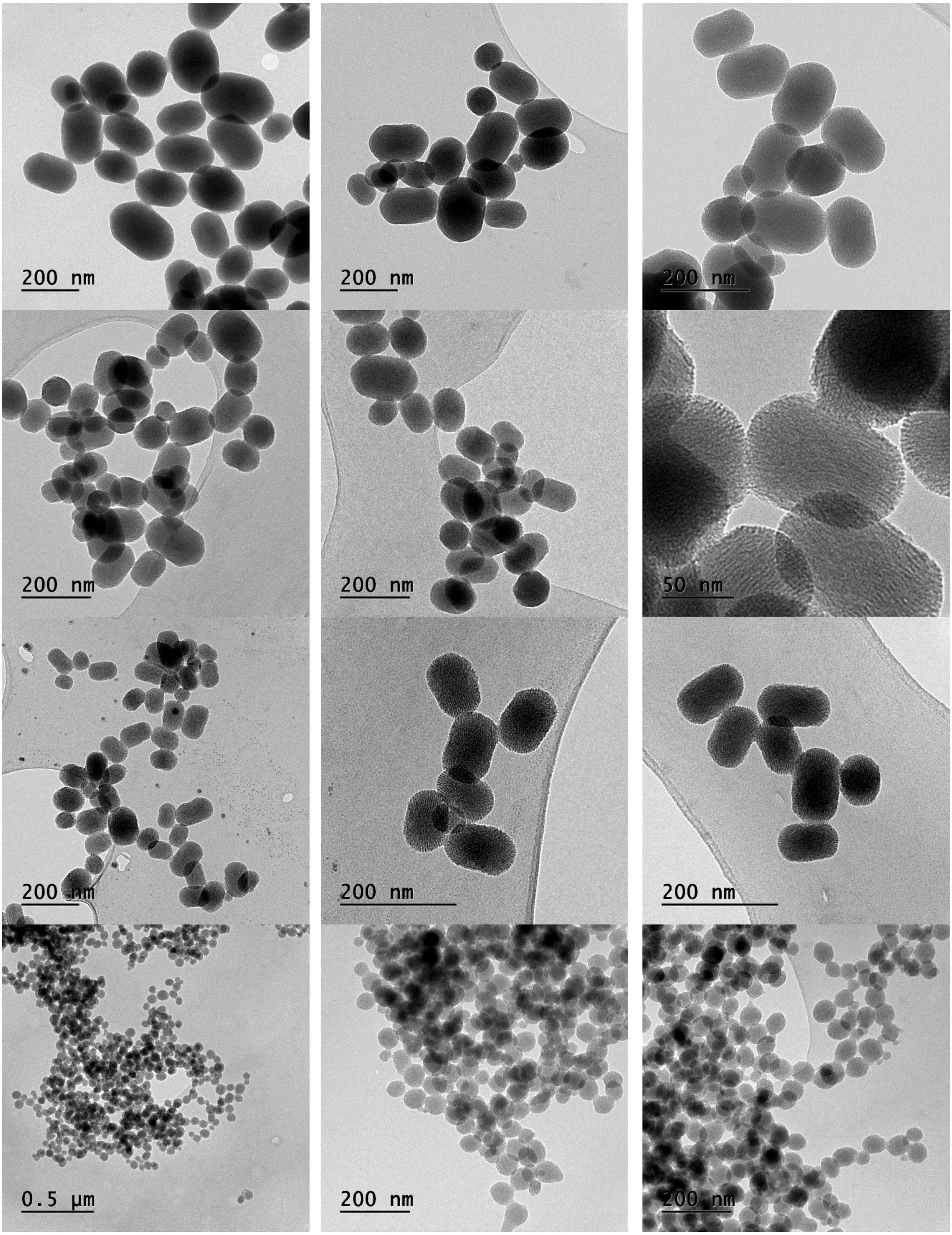

359.2
5 min

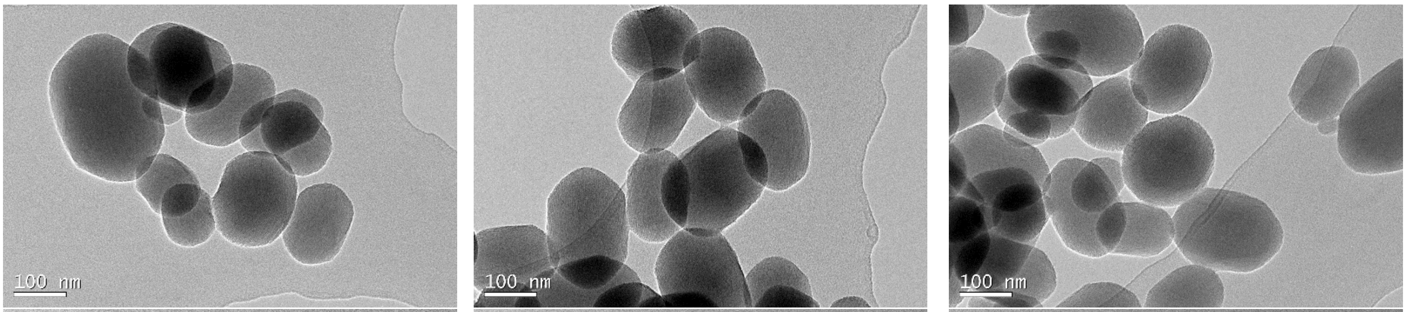

359.2
30 min

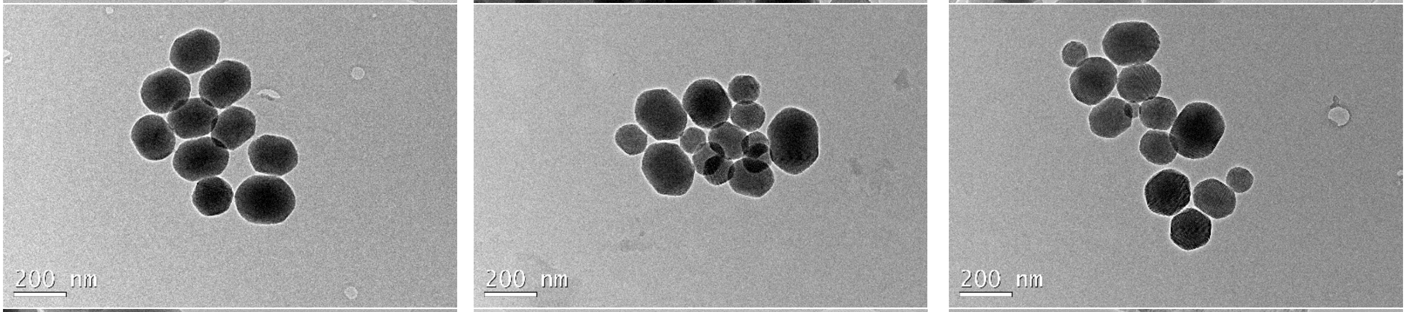

359.2
60 min

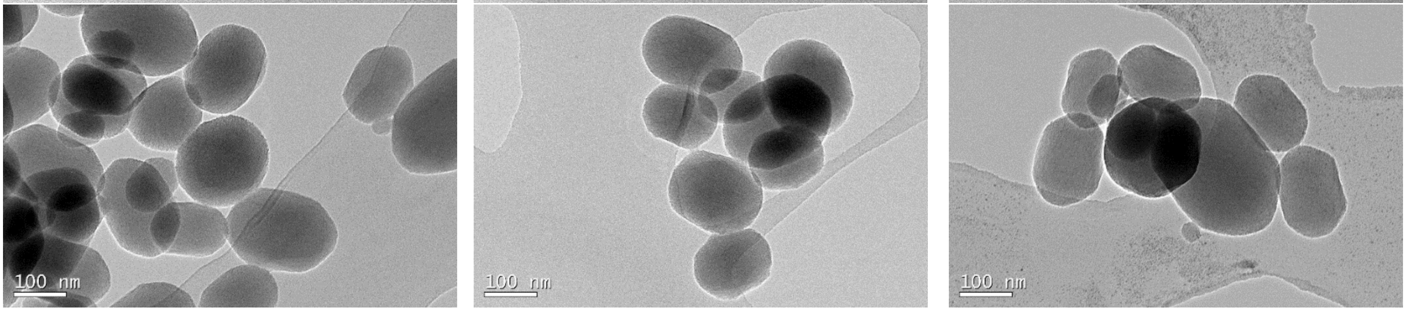

Particle size distribution and Feret diameter obtained from TEM micrographs.

Particle size distribution and frequency histograms obtained from diameter measures obtained from TEM micrographs. Data were collected with ImageJ software and processed with OriginLab2016.

Sample	*Particle size distribution (nm)*	*Feret diameter per particle (nm)*
D3	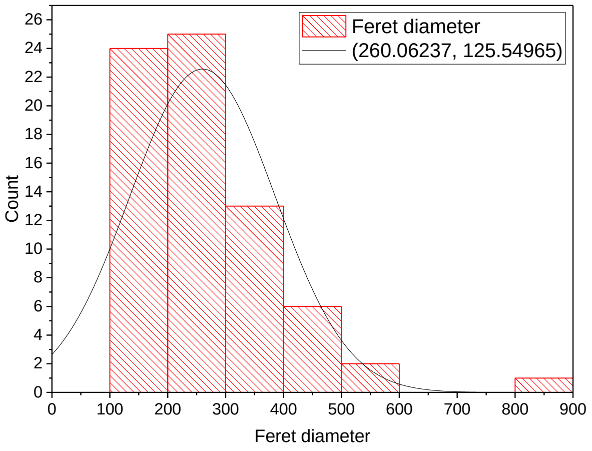	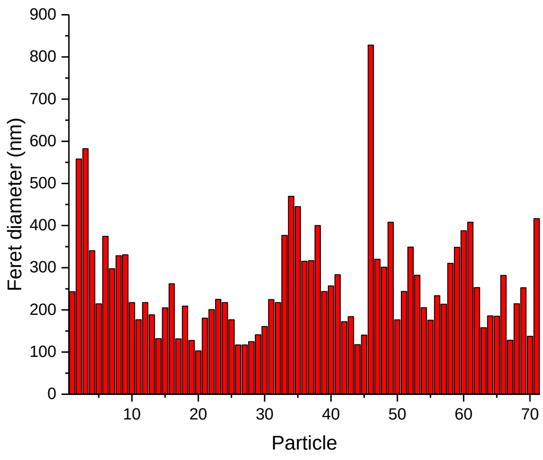

D5

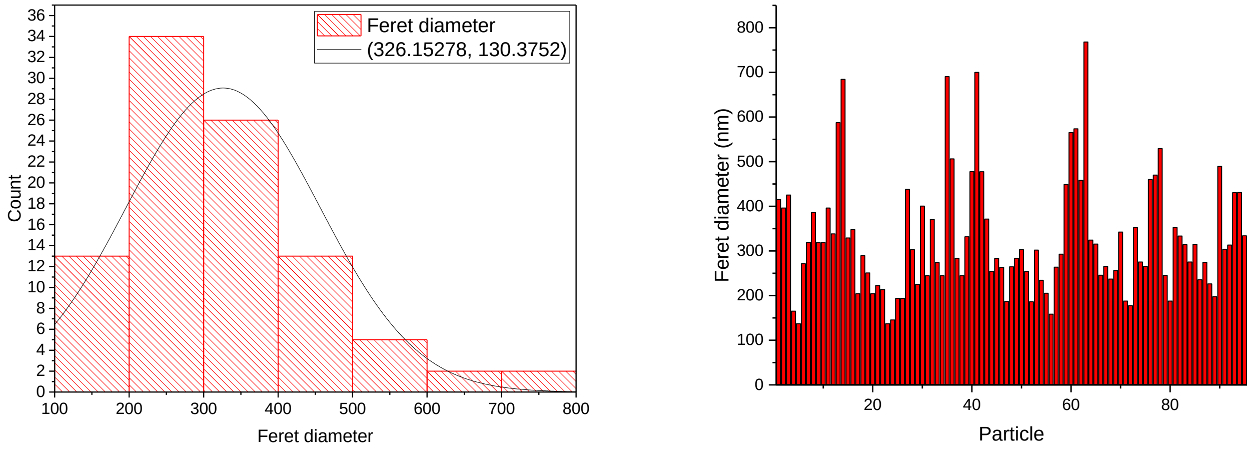
Feret diameter
(326.15278, 130.3752)
Count
Feret diameter
Feret diameter (nm)
Particle

D7

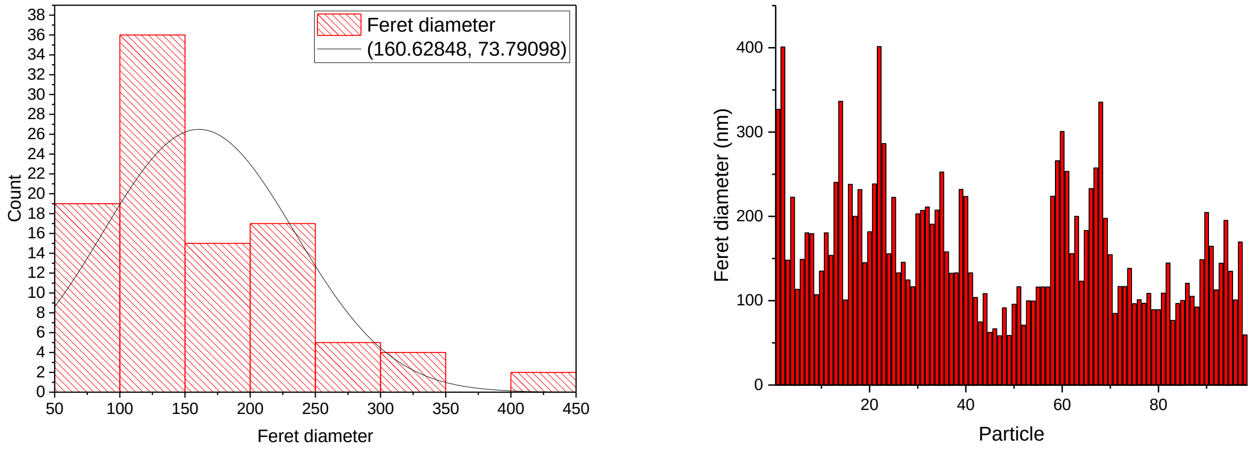
Feret diameter
(160.62848, 73.79098)
Count
Feret diameter
Feret diameter (nm)
Particle

C1

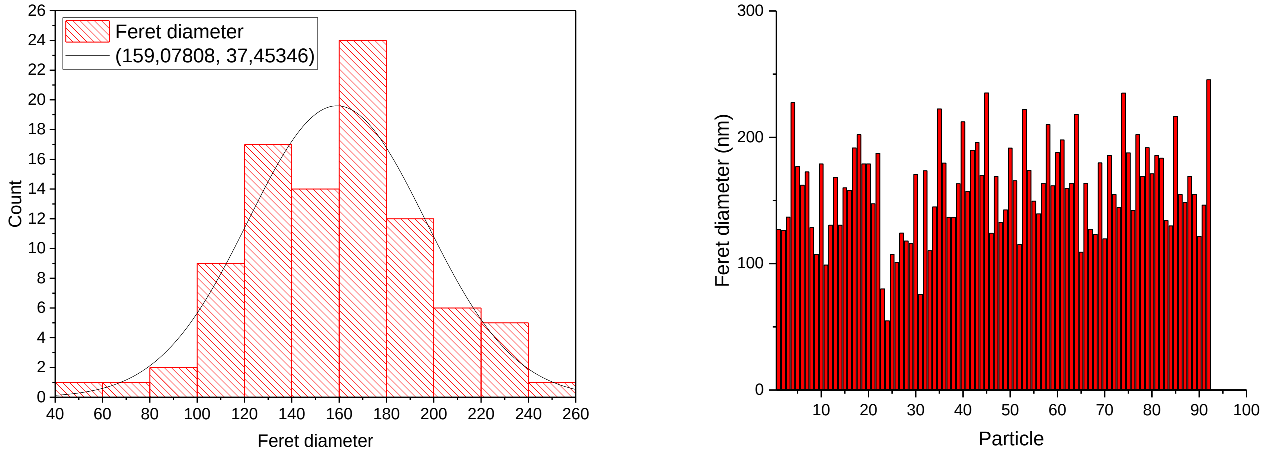
Feret diameter
(159,07808, 37,45346)
Count
Feret diameter
Feret diameter (nm)
Particle

C2

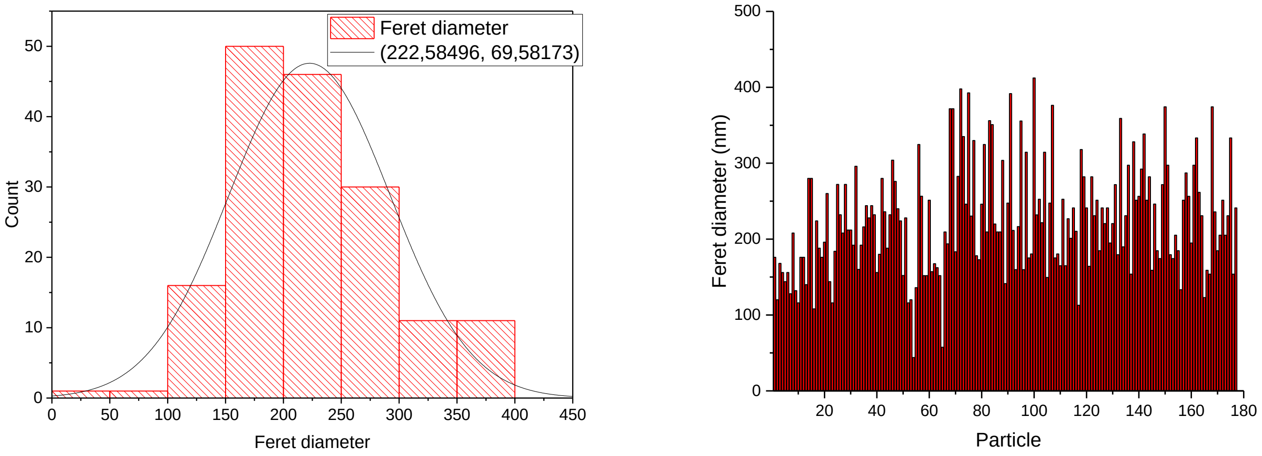

Feret diameter
(222,58496, 69,58173)
Count
Feret diameter
0
10
20
30
40
50
0
50
100
150
200
250
300
350
400
450
Feret diameter (nm)
Particle
0
100
200
300
400
500
20
40
60
80
100
120
140
160
180

C3

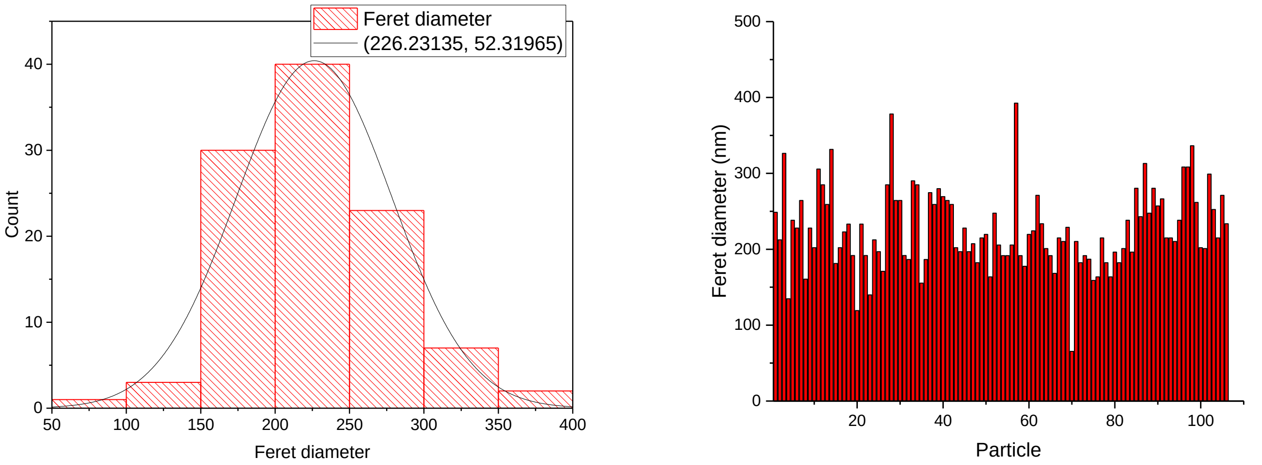

Feret diameter
(226.23135, 52.31965)
Count
Feret diameter
0
10
20
30
40
50
100
150
200
250
300
350
400
Feret diameter (nm)
Particle
0
100
200
300
400
500
20
40
60
80
100

C5

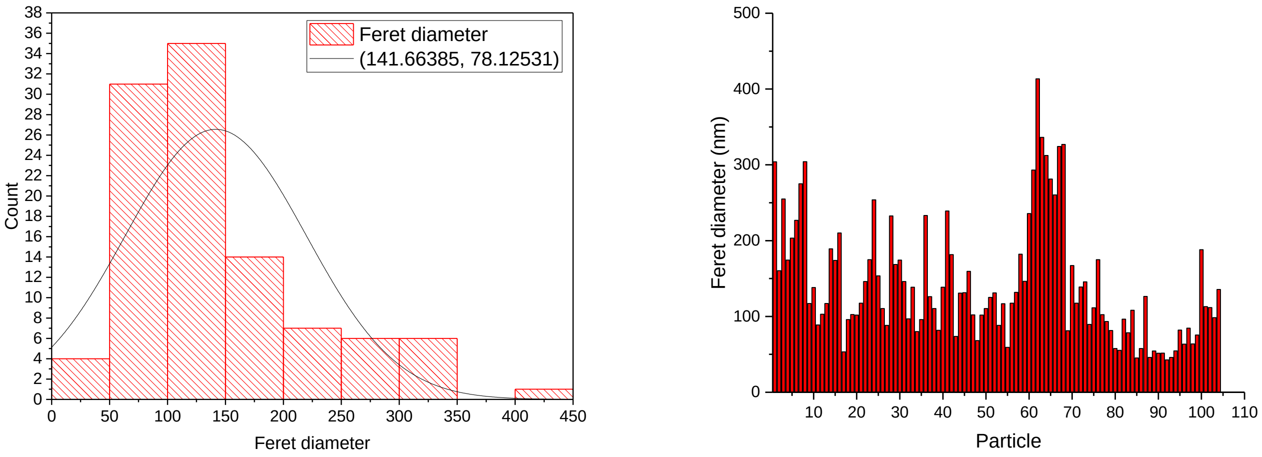

Feret diameter
(141.66385, 78.12531)
Count
Feret diameter
0
2
4
6
8
10
12
14
16
18
20
22
24
26
28
30
32
34
36
38
0
50
100
150
200
250
300
350
400
450
Feret diameter (nm)
Particle
0
100
200
300
400
500
10
20
30
40
50
60
70
80
90
100
110

C6

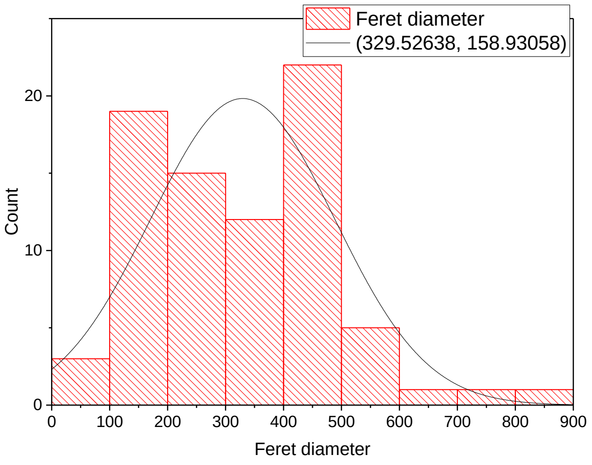
Feret diameter
(329.52638, 158.93058)
Count
0
10
20
0 100 200 300 400 500 600 700 800 900
Feret diameter

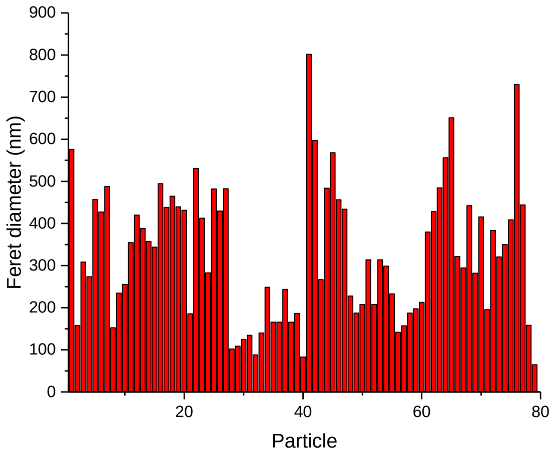
Feret diameter (nm)
0 100 200 300 400 500 600 700 800 900
20 40 60 80
Particle

B1

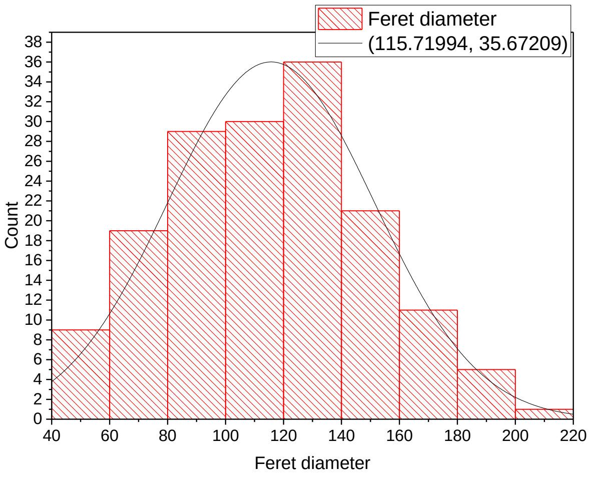
Feret diameter
(115.71994, 35.67209)
Count
0 2 4 6 8 10 12 14 16 18 20 22 24 26 28 30 32 34 36 38
40 60 80 100 120 140 160 180 200 220
Feret diameter

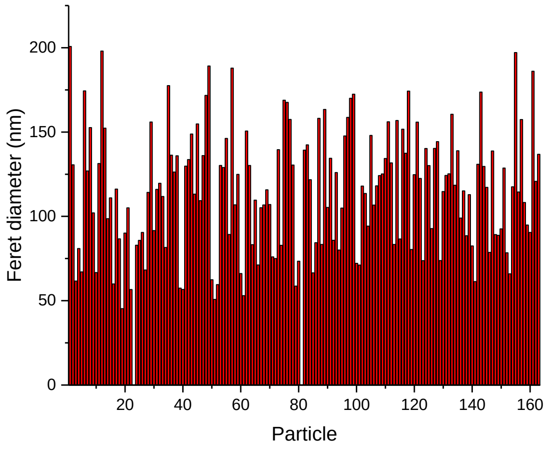
Feret diameter (nm)
0 50 100 150 200
20 40 60 80 100 120 140 160
Particle

B2

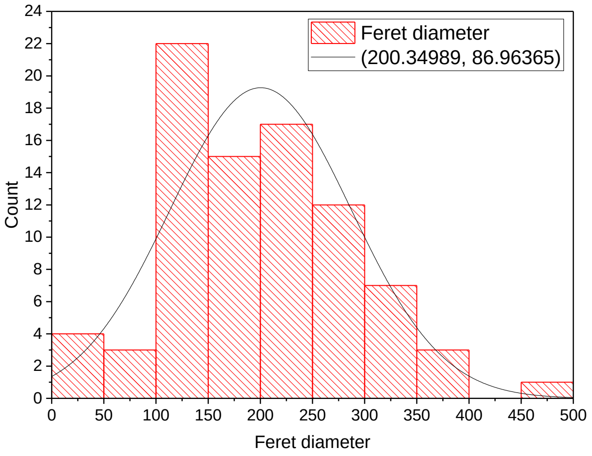
Feret diameter
(200.34989, 86.96365)
Count
0 2 4 6 8 10 12 14 16 18 20 22 24
0 50 100 150 200 250 300 350 400 450 500
Feret diameter

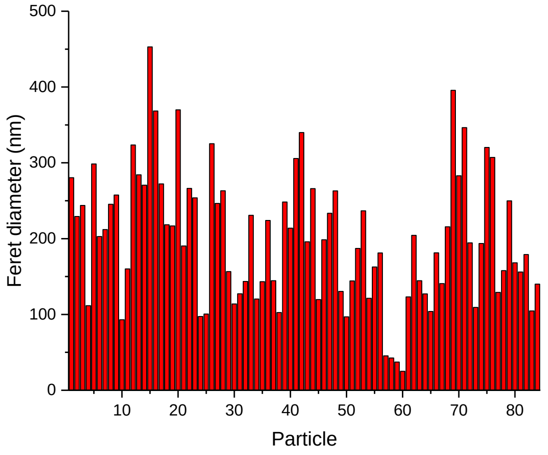
Feret diameter (nm)
0 100 200 300 400 500
10 20 30 40 50 60 70 80
Particle

B3

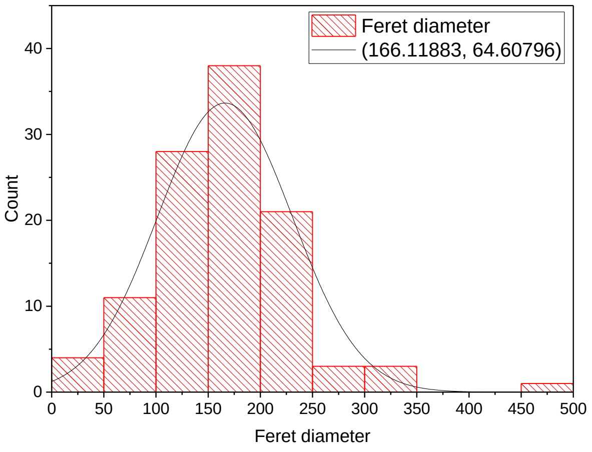
Feret diameter
(166.11883, 64.60796)
Count
Feret diameter

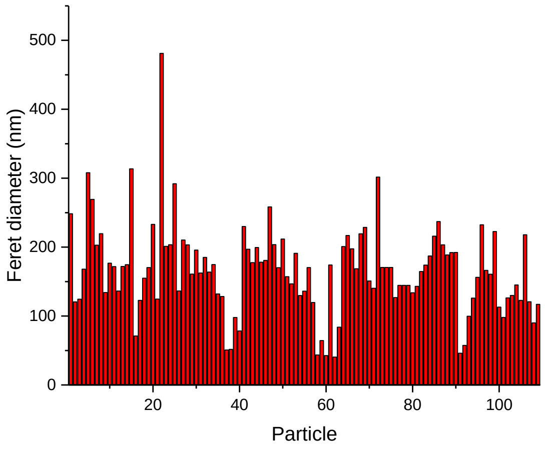
Feret diameter (nm)
Particle

B4

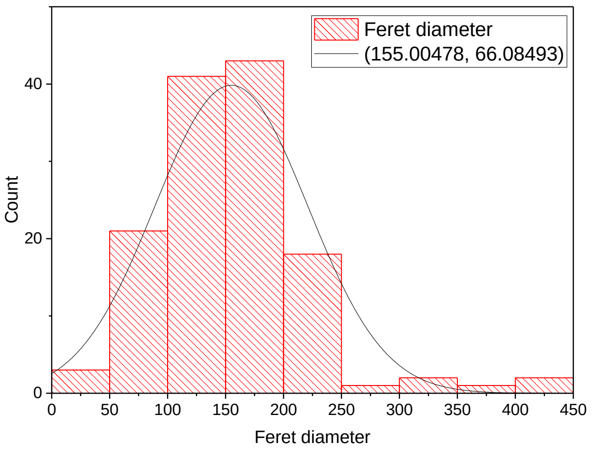
Feret diameter
(155.00478, 66.08493)
Count
Feret diameter

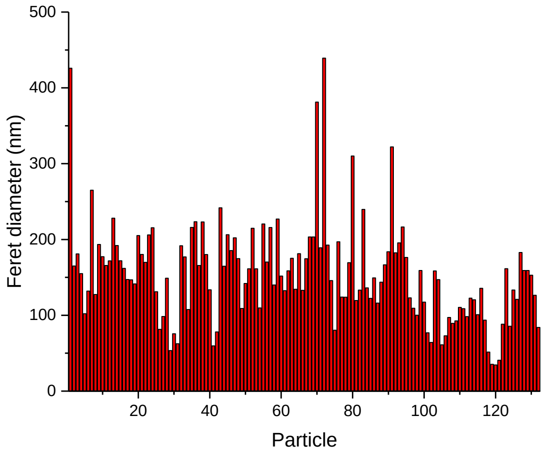
Feret diameter (nm)
Particle

345.2

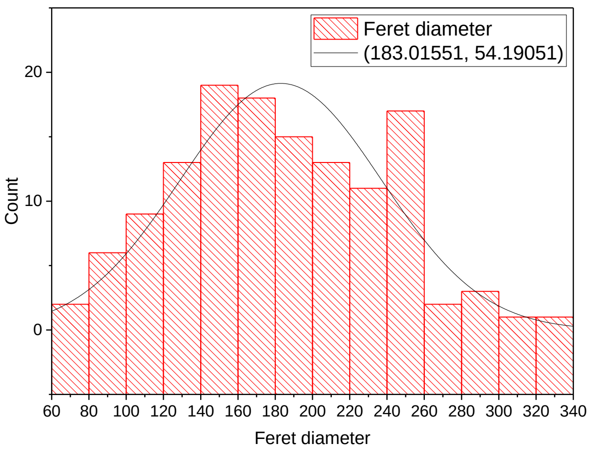
Feret diameter
(183.01551, 54.19051)
Count
Feret diameter

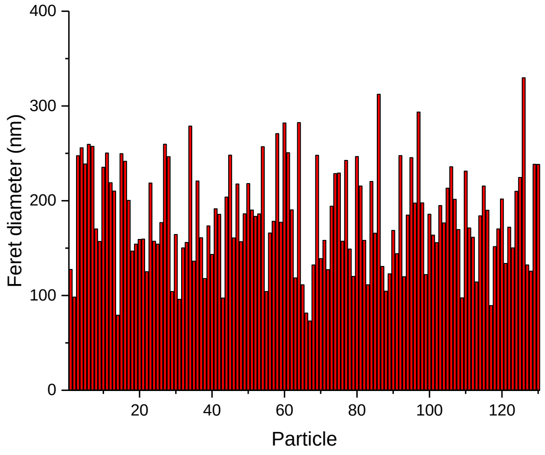
Feret diameter (nm)
Particle

345.4

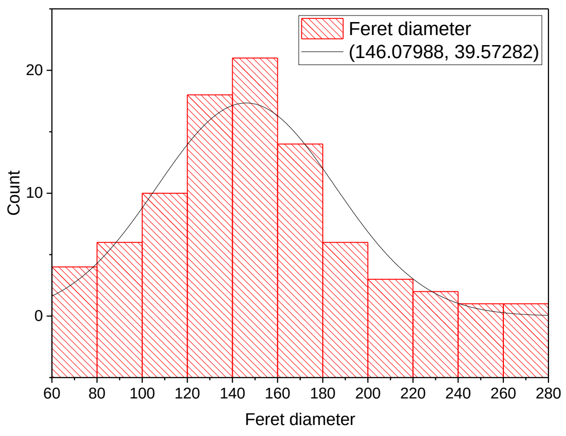

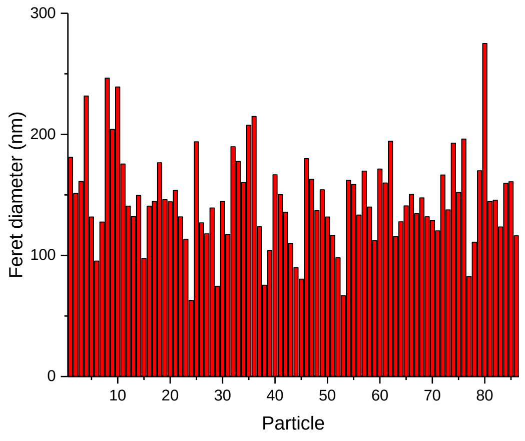

389.1

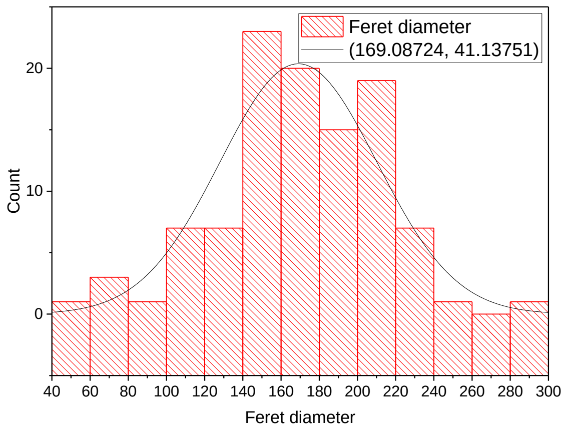

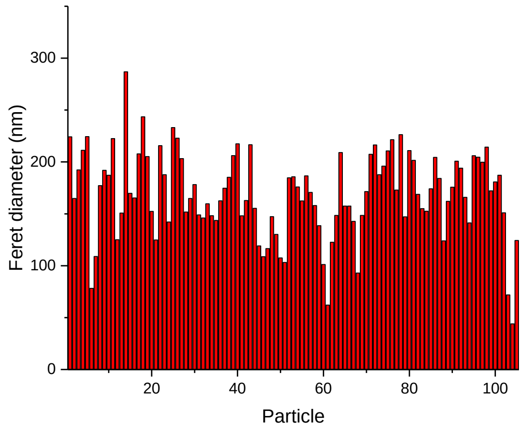

345.3

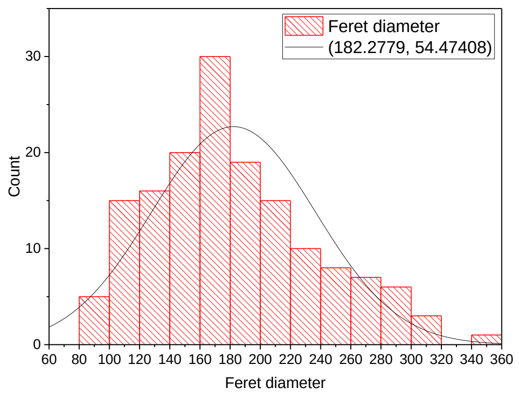

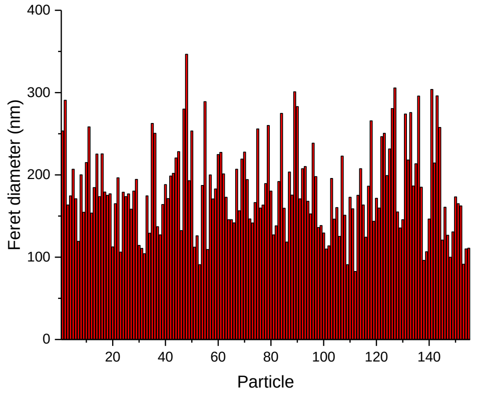

349.2

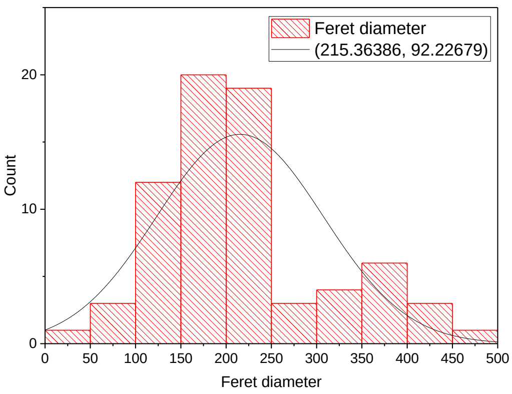

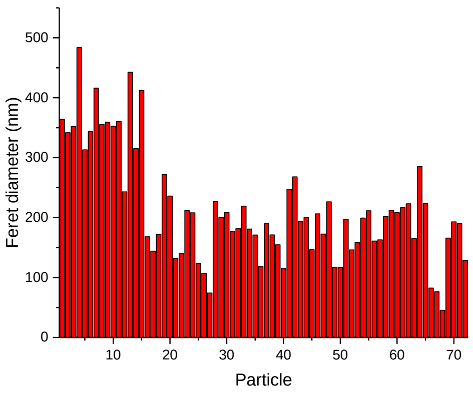

349.1

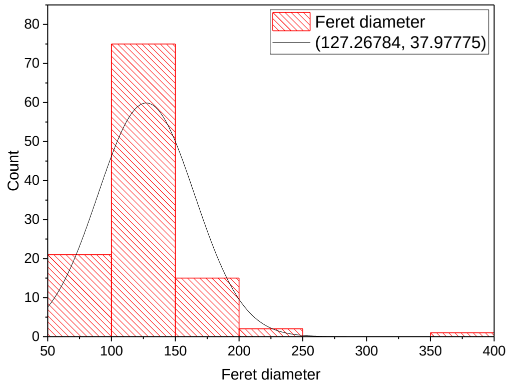

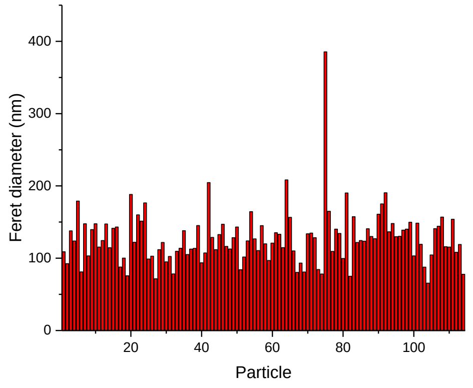

345.7

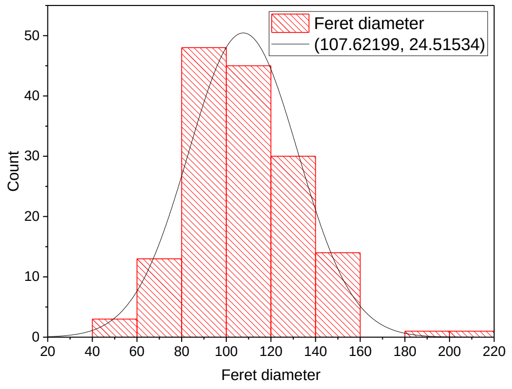

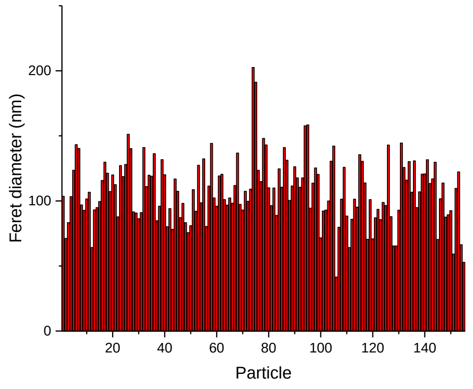

359.2

5 min

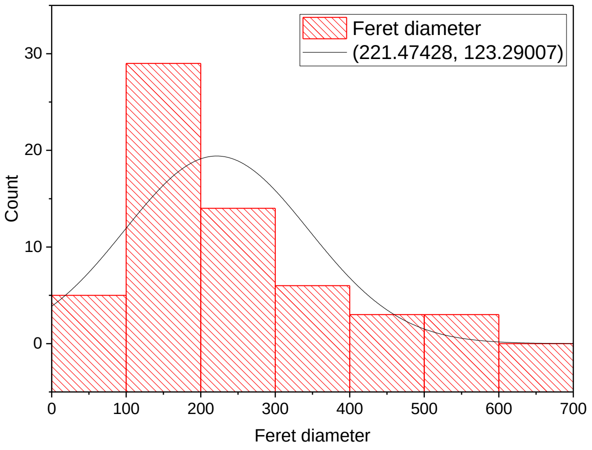

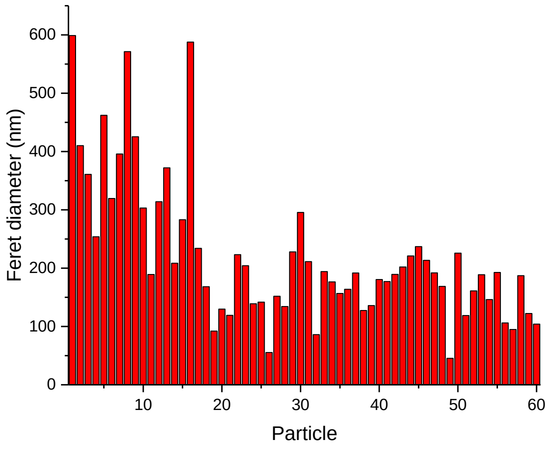

359.2

30 min

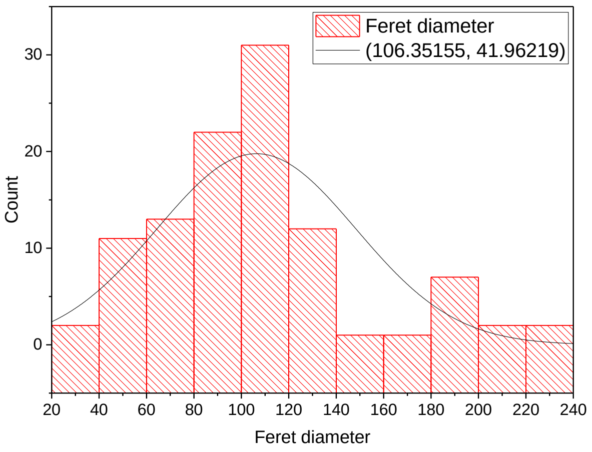

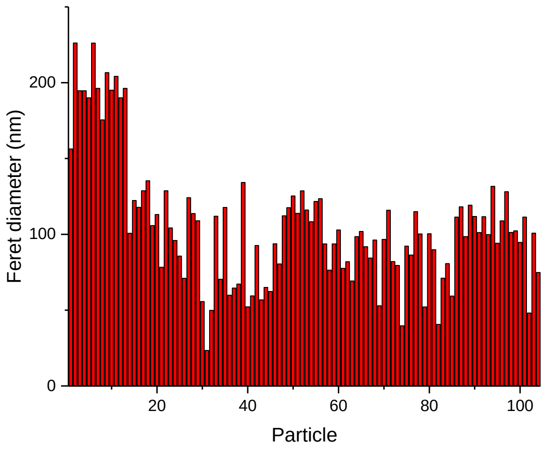

359.2

60 min

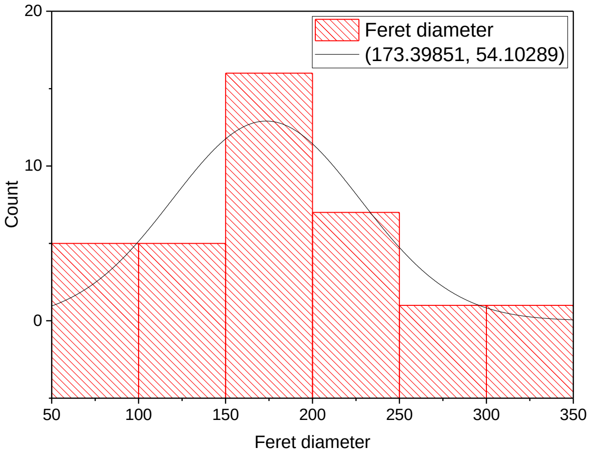

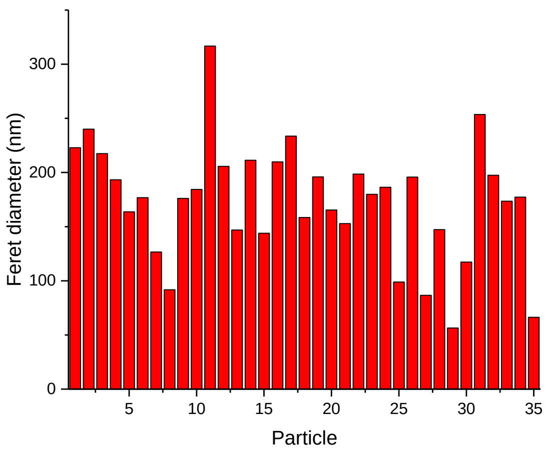

__Infrared spectra for extracted MCM-41 MSNs.__

The analysis of IR vibrational bands was measured on extracted materials and showed the typical bands associated to Si-O bonds. However, in some cases there could be observed vibrational bands associated to the presence of entrapped CTAB within the structure, which points out the existence of obstructed mesopores (D4-D7) that do permit the extraction of the surfactant. The IR spectra were recorded in a Nicolet Nexus (Thermo Scientific) spectrometer equipped with a Smart Golden Gate ATR accessory and placing the sample directly onto the measurement window to reduce the generation of artefacts or manipulation errors.

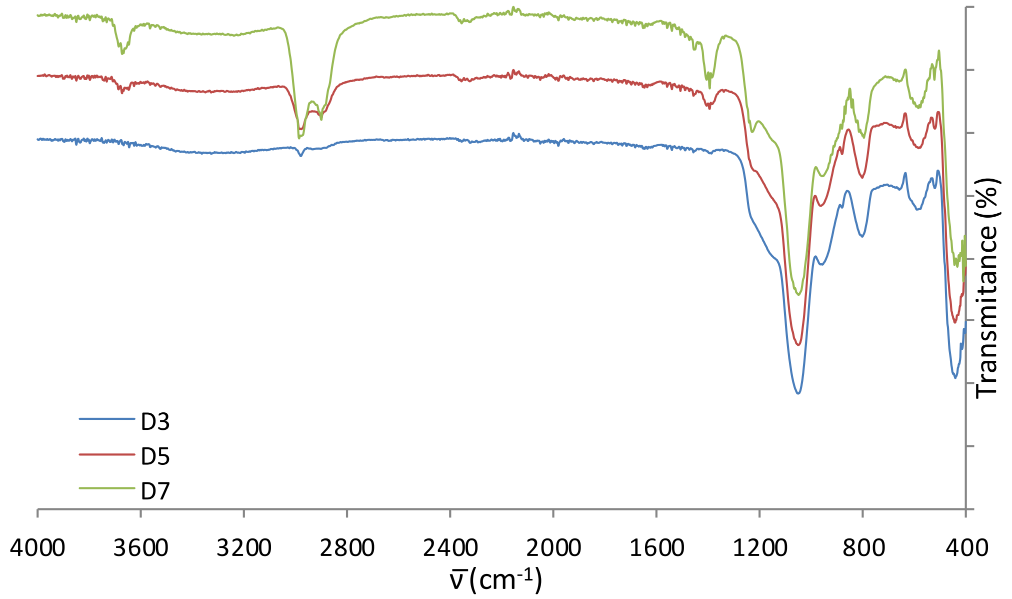

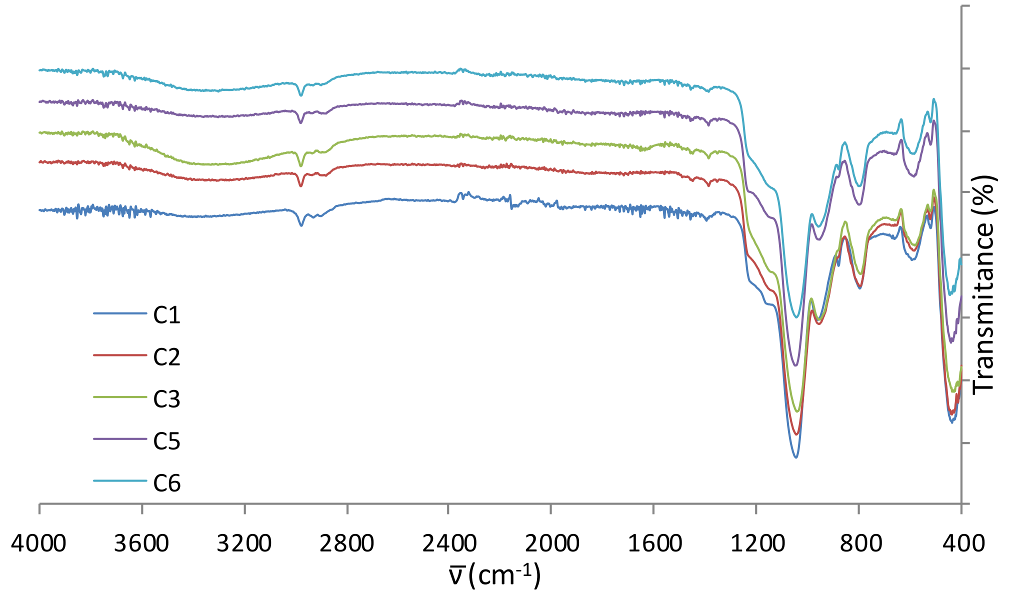

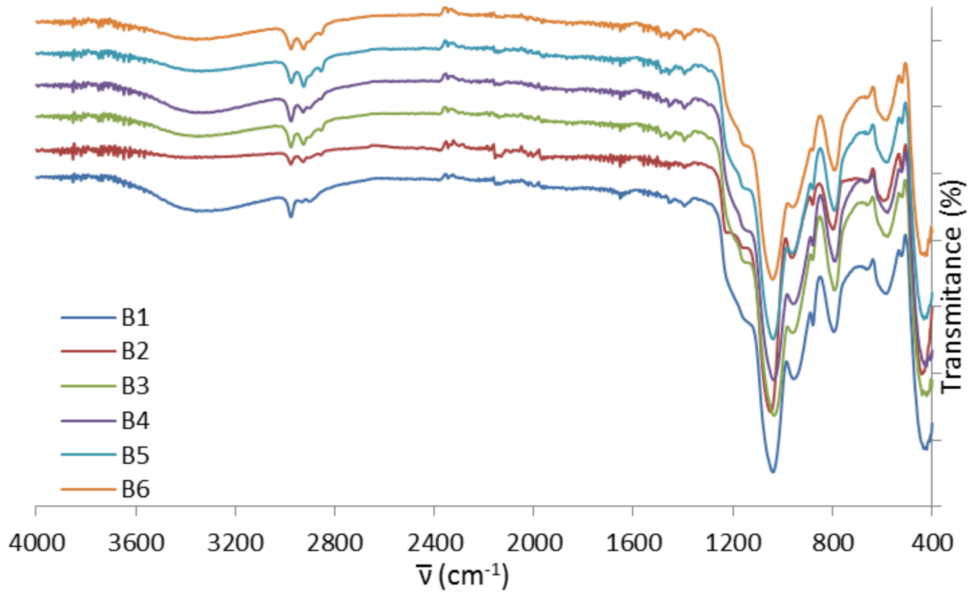
B1
B2
B3
B4
B5
B6
Transmitance (%)
4000
3600
3200
2800
2400
2000
1600
1200
800
400
ν̅ (cm-1)

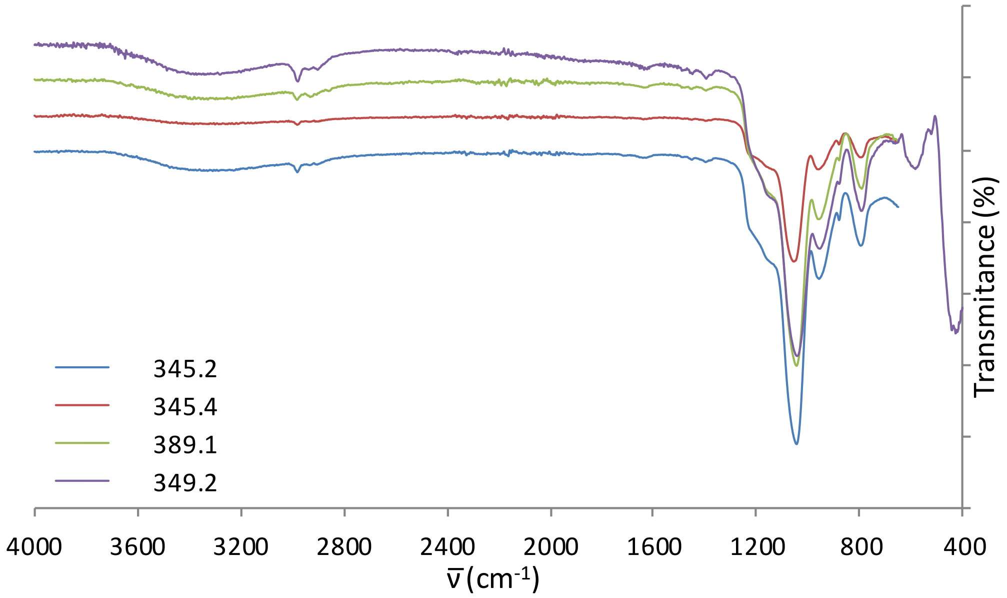
345.2
345.4
389.1
349.2
Transmitance (%)
4000
3600
3200
2800
2400
2000
1600
1200
800
400
ν̅ (cm-1)

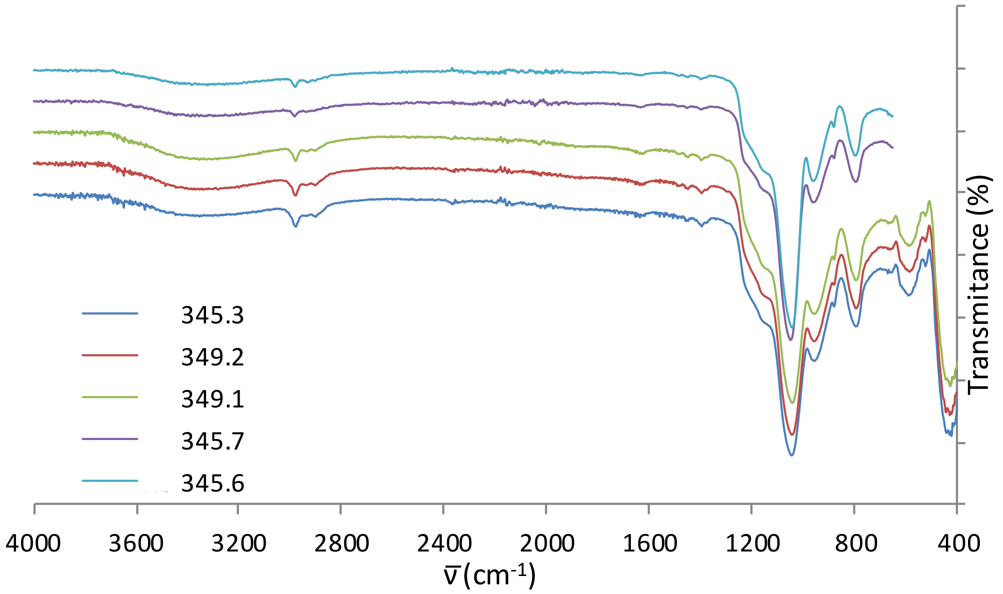
345.3
349.2
349.1
345.7
345.6
Transmitance (%)
4000
3600
3200
2800
2400
2000
1600
1200
800
400
ν̅ (cm-1)

Small Angle X-ray diffraction spectra.

SAXS patterns of prepared nanoparticles. The spectra show the typical long-range ordering diffraction pattern for MCM-41 mesoporous silica with the 100, 110 and 200 peaks as representative diffraction peaks in all cases. Measures were recorded from θ to 2θ in a Philips X-Pert MPD diffractometer fitted with a monochromator and a collimator on the diffracted beam employing the following conditions: 2θ = 0.6 to 6.5º, step size= 0.02º, time per step =5s, Cu Kα = 1.54Å. Data showed have been normalized for clarity.

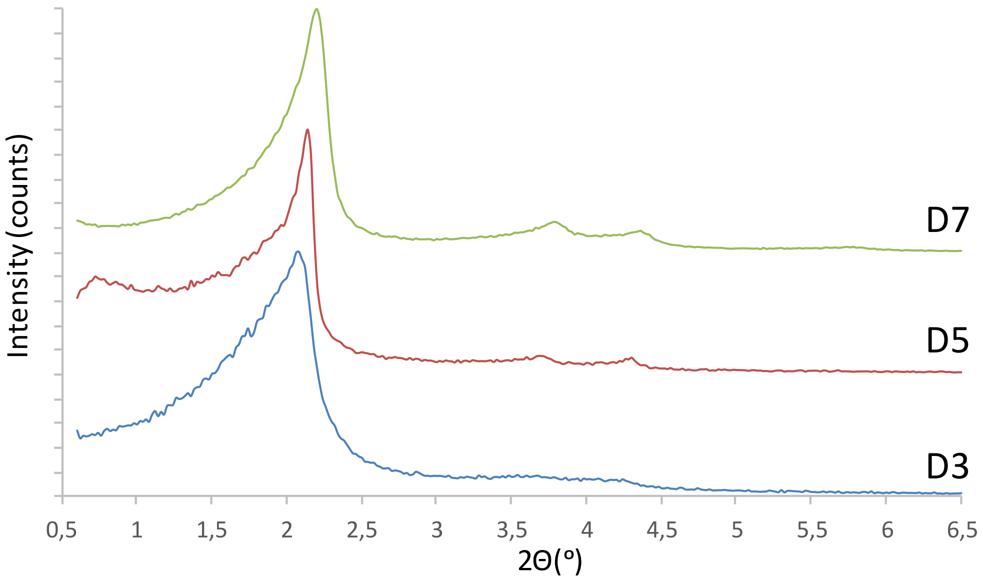

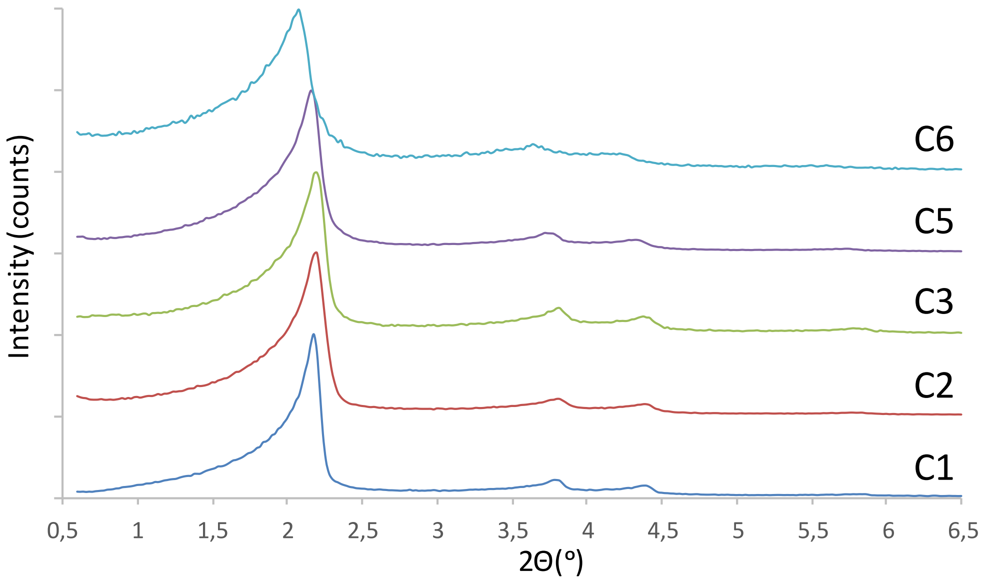

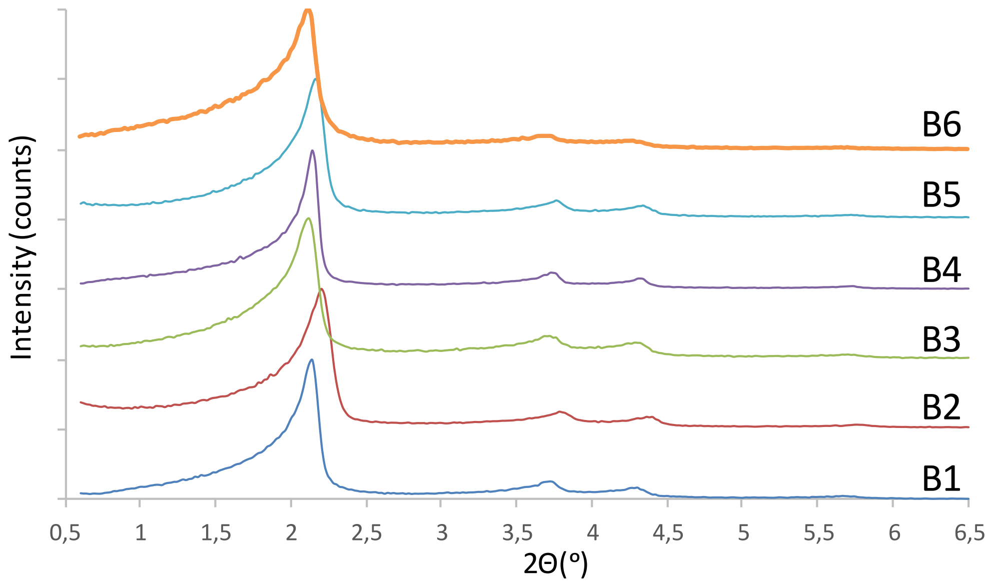
Intensity (counts)
B6
B5
B4
B3
B2
B1
0,5
1
1,5
2
2,5
3
3,5
4
4,5
5
5,5
6
6,5
2Θ(°)

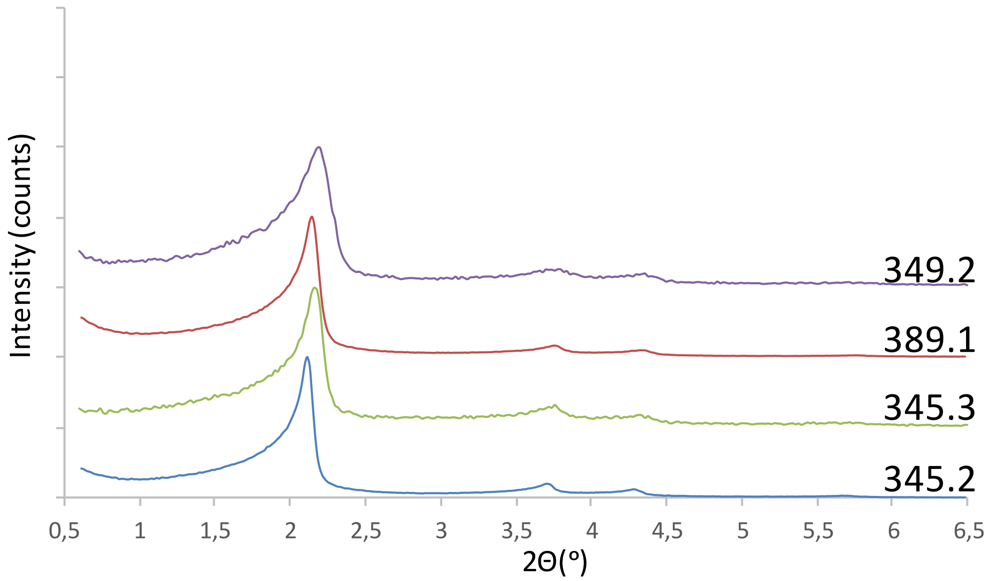
Intensity (counts)
349.2
389.1
345.3
345.2
0,5
1
1,5
2
2,5
3
3,5
4
4,5
5
5,5
6
6,5
2Θ(°)

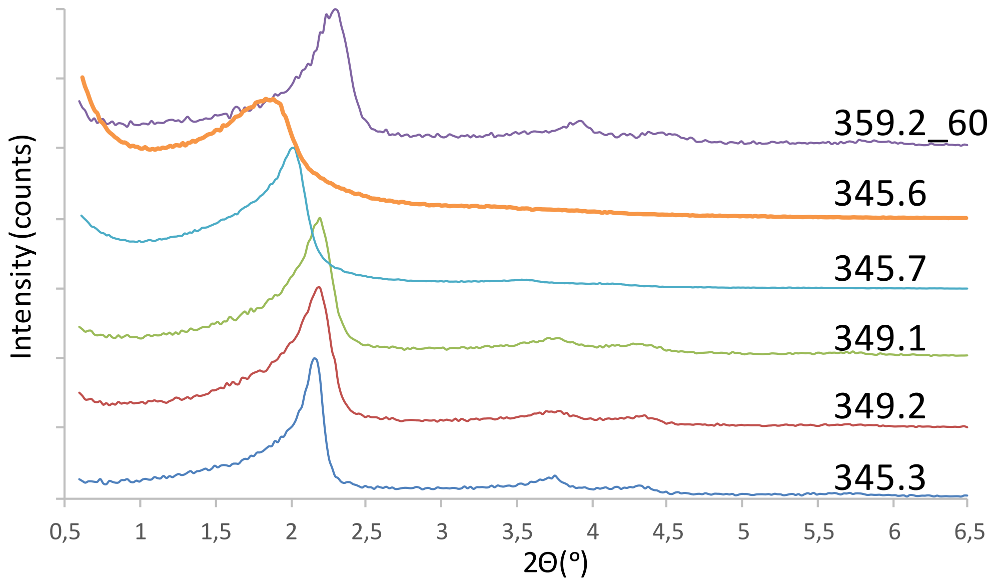
Intensity (counts)
359.2_60
345.6
345.7
349.1
349.2
345.3
0,5
1
1,5
2
2,5
3
3,5
4
4,5
5
5,5
6
6,5
2Θ(°)

N2 adsorption isotherms of MCM-41 MSNs

Pore properties were determined by N_2 adsorption isotherms on a Micromeritics ASAP 2020/3Flex instruments. The surface area was obtained by applying the Brunauer Emmet-Teller (BET) approach to the isotherm. All prepared samples showed the expected type IV N_2 adsorption isotherm typical from the MCM-41 morphology.

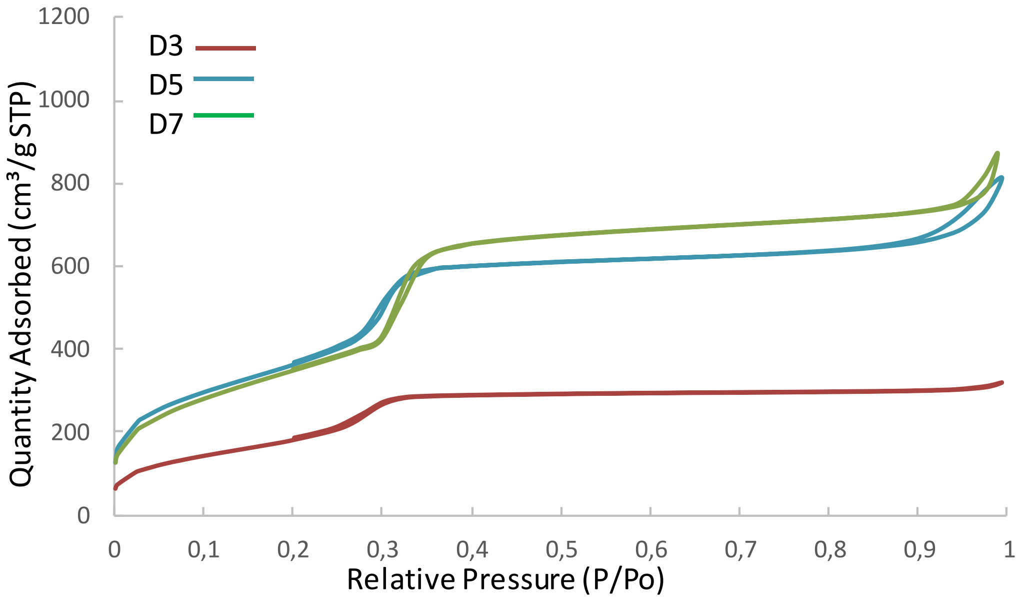

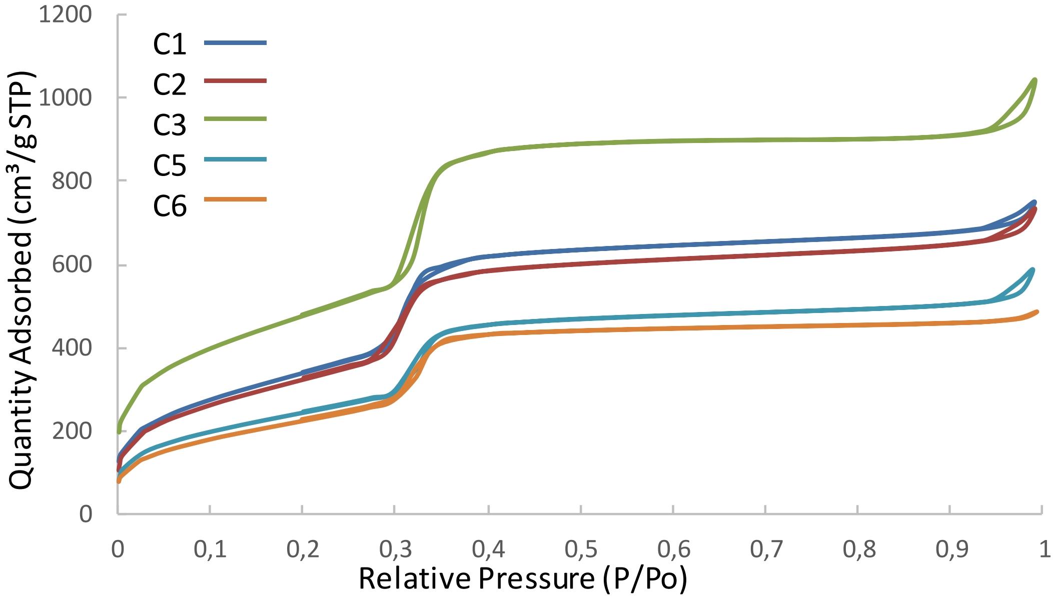

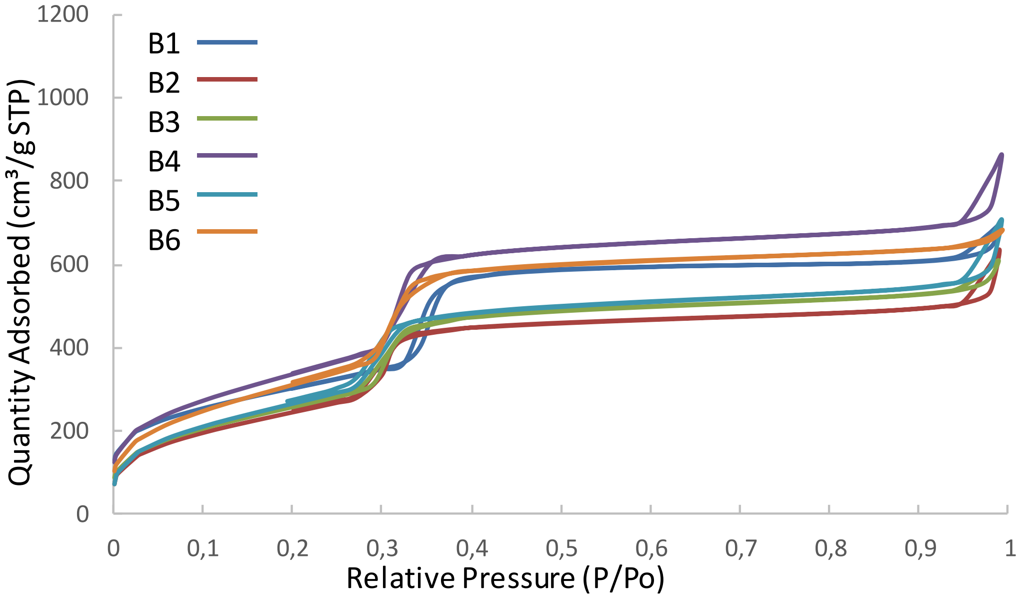
1200
1000
800
600
400
200
0
Quantity Adsorbed (cm³/g STP)
B1
B2
B3
B4
B5
B6
0
0,1
0,2
0,3
0,4
0,5
0,6
0,7
0,8
0,9
1
Relative Pressure (P/Po)

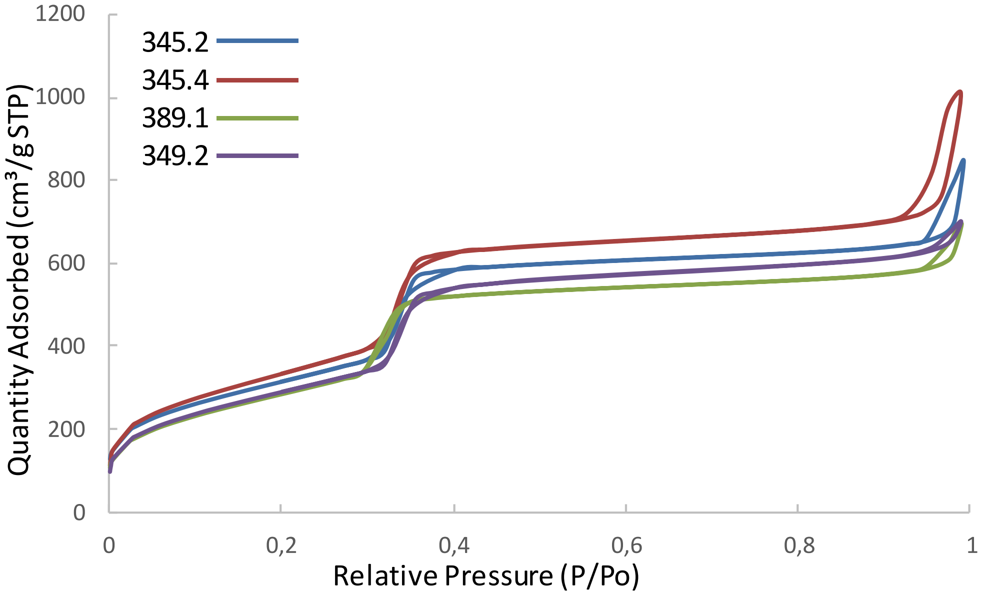
1200
1000
800
600
400
200
0
Quantity Adsorbed (cm³/g STP)
345.2
345.4
389.1
349.2
0
0,2
0,4
0,6
0,8
1
Relative Pressure (P/Po)

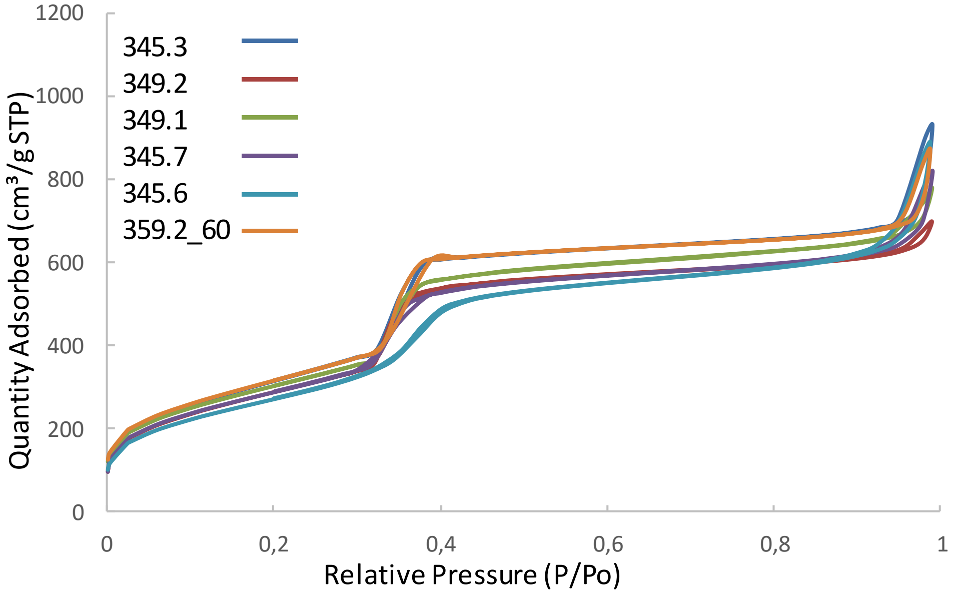
1200
1000
800
600
400
200
0
Quantity Adsorbed (cm³/g STP)
345.3
349.2
349.1
345.7
345.6
359.2_60
0
0,2
0,4
0,6
0,8
1
Relative Pressure (P/Po)

Pore diameters obtained from by N_2 adsorption isotherms of prepared MCM-41 MSNs.

Pore properties were determined by N_2 adsorption isotherms on a Micromeritics ASAP 2020/3Flex instruments. Pore size distribution was obtained using the Barrett–Joyner–Halenda (BJH) approach from the desorption branch of the isotherm and showed mesopores in the range from 2.4 to 3.2 nm typical from the CTAB templated synthesis.

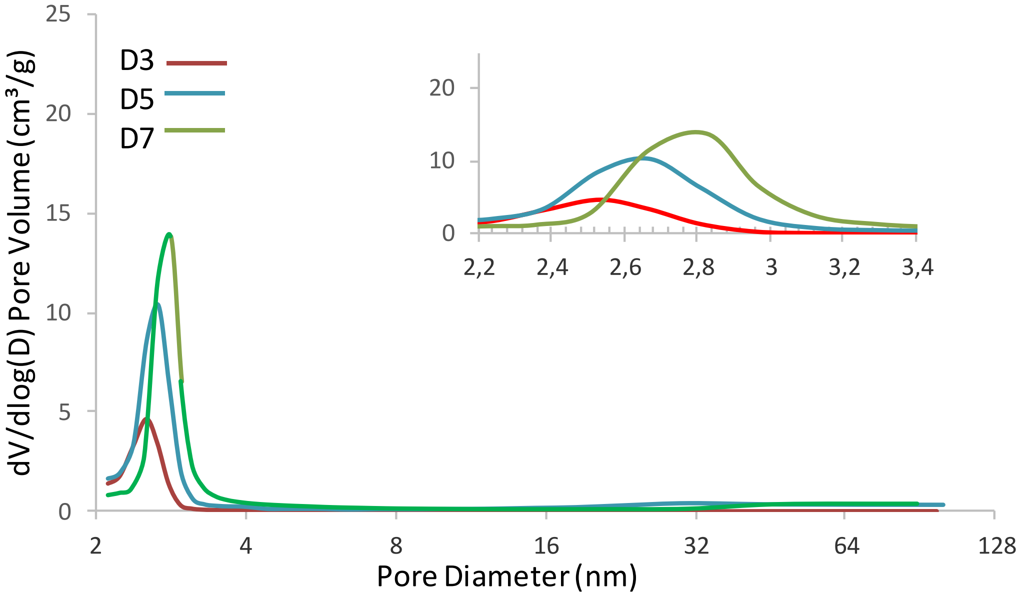

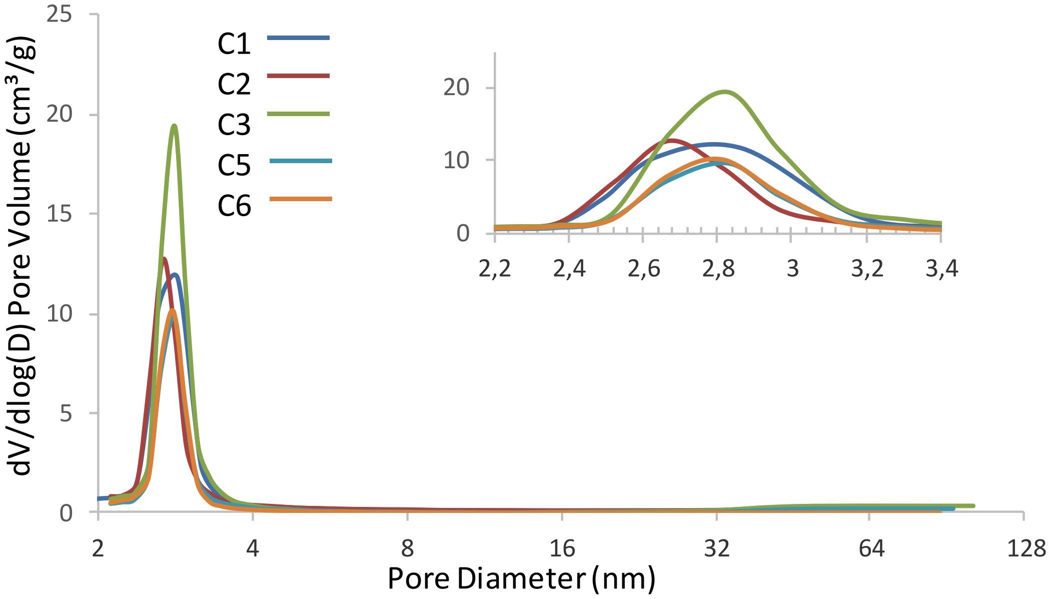

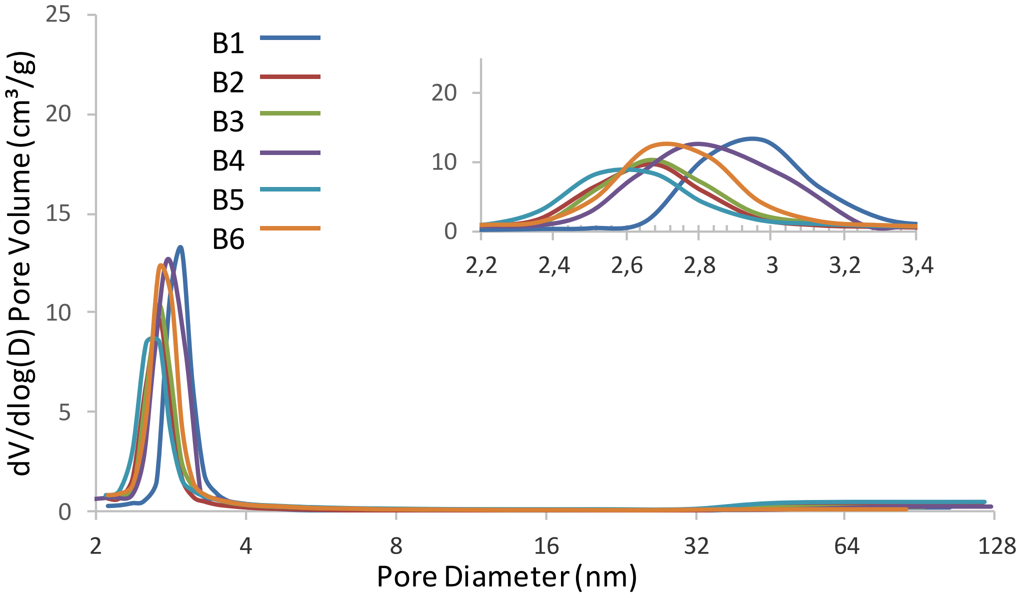
B1
B2
B3
B4
B5
B6
dV/dlog(D) Pore Volume (cm³/g)
Pore Diameter (nm)
25
20
15
10
5
0
2
4
8
16
32
64
128
2,2
2,4
2,6
2,8
3
3,2
3,4

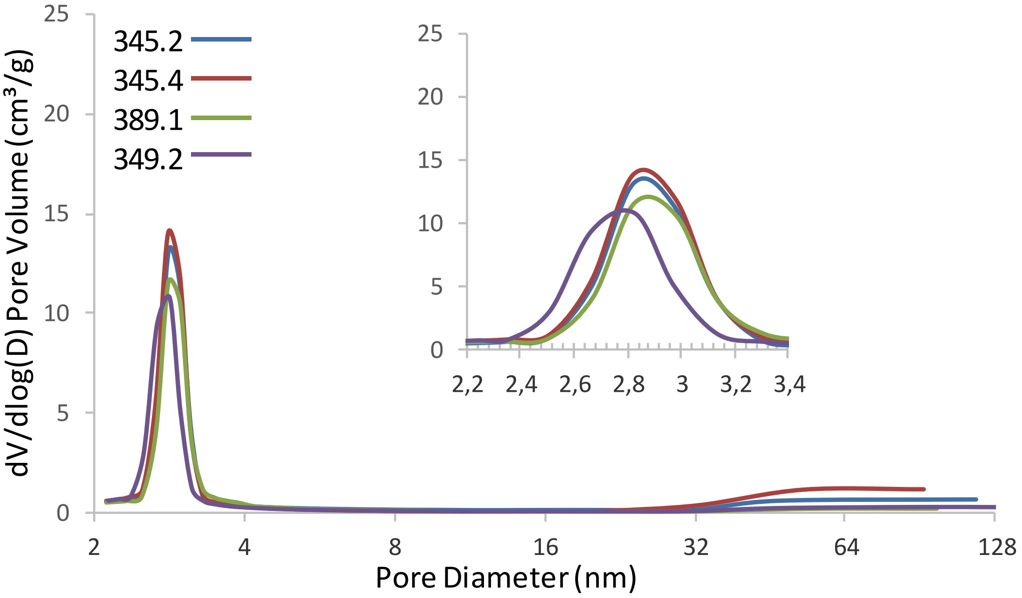
345.2
345.4
389.1
349.2
dV/dlog(D) Pore Volume (cm³/g)
Pore Diameter (nm)
25
20
15
10
5
0
2
4
8
16
32
64
128
2,2
2,4
2,6
2,8
3
3,2
3,4

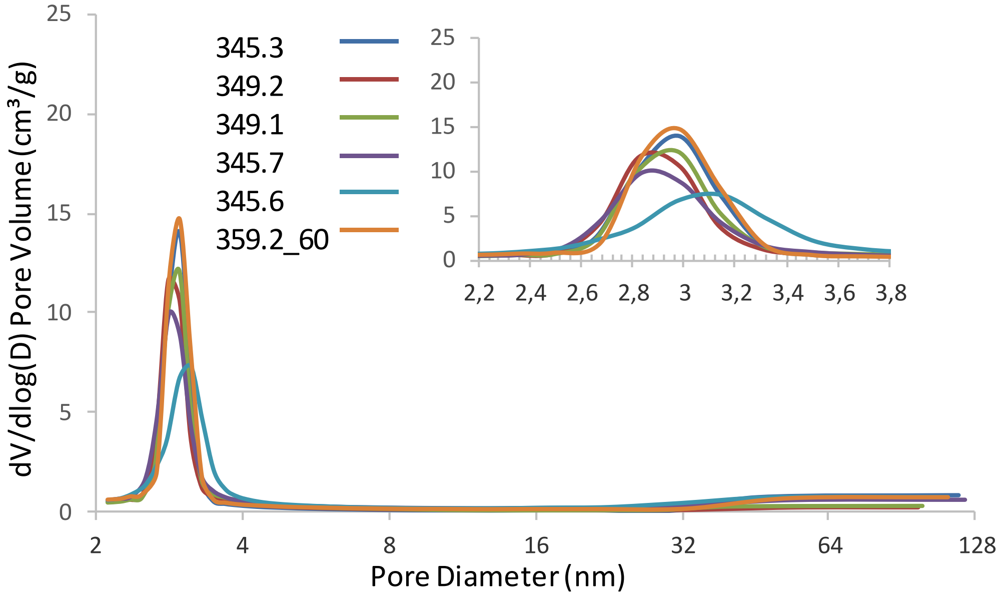
345.3
349.2
349.1
345.7
345.6
359.2_60
dV/dlog(D) Pore Volume (cm³/g)
Pore Diameter (nm)
25
20
15
10
5
0
2
4
8
16
32
64
128
2,2
2,4
2,6
2,8
3
3,2
3,4
3,6
3,8

Calculated surface areas and pore diameters for extracted MCM-41 MSNs

Table S2: Surface area measured by N_2 adsorption isotherm employing the BET model and maximal pore diameter according to BHJ model.

**Sample**	**D3**	**D5**	**D7**	**C1**
Surface area (m^2/g)	754.766±36.751	1467.244±65.887	1339.881±13.366	1304.063±21.785
Pore diameter (nm)	2.532	2.672	2.831	2.867

**Sample**	**C2**	**C3**	**C5**	**C6**
Surface Area (m^2/g)	1254.572±31.701	1739.231±3.007	919.419±6.192	8673.358±11.047
Pore diameter (nm)	2.812	2.827	2.830	2.814

**Sample**	**B1**	**B2**	**B3**	**B4**
Surface Area (m^2/g)	1089.090±2.415	1016.737±65.742	1015.872±24.747	1271.836±7.821
Pore diameter (nm)	2.976	2.675	2.671	2.628

**Sample**	**B5**	**B6**	**345.2**	**345.4**
Surface Area (m^2/g)	1140.733±91.725	1217.344±21.821	1155.670±2.410	1240.488±5.762
Pore diameter (nm)	2.597	2.670	2.827	2.820

**Sample**	**389.1**	**345.3**	**349.2**	**349.1**
Surface Area (m^2/g)	1077.442±8.731	1180.438±4.086	1080.580±2.884	1116.200±2.101
Pore diameter (nm)	2.830	2.979	2.825	2.976

**Sample**	**345.7**	**345.6**	**359.2_60**	
Surface Area (m^2/g)	1071.048±3.811	1025.492±7.056	1171.274±3.786	
Pore diameter (nm)	2.837	3.150	2.982	
